# Multiple regulatory variants located in cell type-specific enhancers within the *PKP2* locus form major risk and protective haplotypes for canine atopic dermatitis in German shepherd dogs

**DOI:** 10.1186/s12863-016-0404-3

**Published:** 2016-06-29

**Authors:** Katarina Tengvall, Sergey Kozyrev, Marcin Kierczak, Kerstin Bergvall, Fabiana H. G. Farias, Brita Ardesjö-Lundgren, Mia Olsson, Eva Murén, Ragnvi Hagman, Tosso Leeb, Gerli Pielberg, Åke Hedhammar, Göran Andersson, Kerstin Lindblad-Toh

**Affiliations:** Science for Life Laboratory, Department of Medical Biochemistry and Microbiology, Uppsala University, Uppsala, Sweden; Department of Clinical Sciences, Swedish University of Agricultural Sciences, Uppsala, Sweden; Department of Animal Breeding and Genetics, Swedish University of Agricultural Sciences, Uppsala, Sweden; Department of Medicine, Rheumatology Unit, Karolinska Institute, Stockholm, Sweden; Institute of Genetics, University of Bern, Bern, Switzerland; Broad Institute of MIT and Harvard, Cambridge, MA USA

**Keywords:** *PKP2*, Atopic dermatitis, Genetic association, Luciferase reporter assay, Cell type-specific enhancers, Dog, Plakophilin 2, Eczema

## Abstract

**Background:**

Canine atopic dermatitis (CAD) is a chronic inflammatory skin disease triggered by allergic reactions involving IgE antibodies directed towards environmental allergens. We previously identified a ~1.5 Mb locus on canine chromosome 27 associated with CAD in German shepherd dogs (GSDs). Fine-mapping indicated association closest to the *PKP2* gene encoding plakophilin 2.

**Results:**

Additional genotyping and association analyses in GSDs combined with control dogs from five breeds with low-risk for CAD revealed the top SNP 27:19,086,778 (*p* = 1.4 × 10^−7^) and a rare ~48 kb risk haplotype overlapping the *PKP2* gene and shared only with other high-risk CAD breeds. We selected altogether nine SNPs (four top-associated in GSDs and five within the ~48 kb risk haplotype) that spanned ~280 kb forming one risk haplotype carried by 35 % of the GSD cases and 10 % of the GSD controls (OR = 5.1, *p* = 5.9 × 10^−5^), and another haplotype present in 85 % of the GSD cases and 98 % of the GSD controls and conferring a protective effect against CAD in GSDs (OR = 0.14, *p* = 0.0032). Eight of these SNPs were analyzed for transcriptional regulation using reporter assays where all tested regions exerted regulatory effects on transcription in epithelial and/or immune cell lines, and seven SNPs showed allelic differences. The DNA fragment with the top-associated SNP 27:19,086,778 displayed the highest activity in keratinocytes with 11-fold induction of transcription by the risk allele versus 8-fold by the control allele (p_difference_ = 0.003), and also mapped close (~3 kb) to an ENCODE skin-specific enhancer region.

**Conclusions:**

Our experiments indicate that multiple CAD-associated genetic variants located in cell type-specific enhancers are involved in gene regulation in different cells and tissues. No single causative variant alone, but rather multiple variants combined in a risk haplotype likely contribute to an altered expression of the *PKP2* gene, and possibly nearby genes, in immune and epithelial cells, and predispose GSDs to CAD.

**Electronic supplementary material:**

The online version of this article (doi:10.1186/s12863-016-0404-3) contains supplementary material, which is available to authorized users.

## Background

Canine atopic dermatitis (CAD) is defined as an inflammatory and pruritic allergic skin disease with a genetic predisposition and where the development is influenced by environmental factors [1, 2]. The symptoms of CAD include eczematous skin predominantly in the flex and friction areas of the body [3], very similar to atopic dermatitis (AD) in humans [4, 5]. The immune response is primarily due to immunoglobulin E (IgE) antibodies recognizing harmless environmental allergens, resulting in a degranulation of active mediators, such as histamine, by mast cells and eosinophils. The overall prevalence of CAD in dogs has been difficult to estimate, but reports typically range from 3–15 % [6, 7]. Genetic factors are likely to play a substantial role in CAD as it is highly overrepresented in certain high-risk (HRCAD) dog breeds including Boxer, Bull terrier, West Highland white terrier (WHWT), German shepherd dog (GSD), Labrador retriever (LR) [8–11], and Golden retriever (GR) [10, 12]. Face, ears (otitis externa), paws, extremities, ventrum, and flex-zones are typically affected by pruritus and erythema [3]. The affected body-regions seem to differ between breeds. GSDs for example are typically affected by otitis externa and eczema of the belly/groin, whereas Boxers are predisposed to facial area symptoms [10].

Several genetic risk factors have been suggested to contribute to the development of AD in humans [13] and dogs [14]. Genes reported in human AD predominantly fall into two main pathophysiological groups: *i*) immune-mediated pathways and *ii*) skin barrier functions [13]. The most striking findings are mutations in the filaggrin gene (*FLG*) associated with AD [15]. Filaggrin represents one of the proteins that are essential for the cornified envelope of the epidermis [16]. *FLG* mutations lead to an impaired skin barrier, which enhances allergen penetrance and subsequently cutaneous inflammation driven by type 2 T helper (Th2) cells, and to some extent explain AD predisposition in humans. Interestingly, an altered *FLG* mRNA and protein expression has been detected in skin of atopic dogs when compared to skin from healthy control dogs [17]. Moreover, a mutation in the plakophilin 1 gene (*PKP1*) resulting in PKP1 protein deficiency in the skin causes ectodermal dysplasia-skin fragility syndrome in Chesapeake Bay retriever dogs [18]. The same skin fragility disease caused by PKP1 protein loss has been detected also in human [19]. Thus, we anticipate that genetic risk factors identified for CAD may be of importance for human skin diseases such as AD.

In our previous study [20], we identified a ~1.5 Mb risk haplotype on canine chromosome 27 (CFA27) associated with CAD in GSDs (GWAS top SNP 27:19,140,837 on CanFam2.0; p_raw_ = 3.1 × 10^−7^, p_genome_ = 0.03). Targeted re-sequencing and further genotyping in GSDs suggested the strongest association in a ~209 kb region harboring the plakophilin 2 gene (*PKP2*), which was subsequently suggested as the top candidate gene [20]. PKP2 proteins recruit desmoplakin to cell-cell contacts and are crucial for a proper desmosome assembly [21]. Desmosomes are intercellular mechanical junctions that contribute to strength and integrity in tissues such as the myocardium and epidermis that exhibit mechanical stress [22]. Other functions of plakophilins include involvement in multiple signaling and metabolic processes, and also in transcriptional activity (reviewed in [23]). For example, PKP2 binds to β-catenin in the cytoplasm and overexpression of PKP2 has been suggested to reduce the pool of β-catenin available for E-cadherin binding, which may thereby affect cell adhesion [24]. Moreover, PKP2 forms complexes with RNA polymerase III subunits and is generally present in the nuclei of many cell types. Interestingly, in the top layers of skin epithelia PKP2 is excluded from the desmosomes and instead accumulated in the nuclei of the keratinocytes [24]. In addition to *PKP2*, the genes *YARS2* (tyrosyl-tRNA synthetase), *DNM1L* (Dynamin 1-like), and *FGD4* (FYVE, RhoGEF and PH domain containing 4) may potentially be involved in CAD development, due to their location close (~30-150 kb) to the associated locus.

Here, we aimed at scrutinizing the *PKP2* risk locus, pinpointing candidate variants and investigating their functionality. We performed an across-breed analysis that defined one top-associated SNP and a rare 48 kb risk haplotype in GSDs overlapping with regions of high regulatory potential within the *PKP2* gene. Eight candidate SNPs were included in functional evaluation and displayed variable regulatory potential and allelic differences dependent on cell type.

## Results

### Genotyping of SNPs located within the *PKP2* locus

We genotyped in total 381 dogs including GSDs and various other breeds with either high or low risk for CAD (HRCAD or LRCAD breeds; Methods) for 120 SNPs spanning a region of ~1 Mb (see Additional file 1: Table S1 for both CanFam2 and CanFam3.1 SNP positions). The SNPs were in LD with the top-associated GWAS SNPs [20] and selected based on multiple criteria. SNPs that were not present in previously published whole genome sequence data from LRCAD breed pools [25] were regarded as potentially functionally important as well as SNPs located in regulatory regions according to the human UCSC browser. Finally, SNPs were selected to cover the region sufficiently and the previous top SNPs were included as references. For details see Methods. The majority of the genotyped SNPs (N_SNPs_ = 102) were located in a 340 kb region within the *PKP2* locus, including the top-associated region (~209 kb) defined by previous fine-mapping [20]. After quality control, 370 dogs and 104 SNPs (out of which seven were included in previous fine-mapping) remained (Table 1 and Additional file 1: Table S1 and Additional file 2: Table S2).

**Table 1:** Dogs included in genotyping of 120 SNPs

Breed	CAD cases	CAD controls
German shepherd^a^	91	83
Labrador retriever^a^	21	14
Golden retriever^a^	10	15
West Highland white terrier^a^	18	15
Boxer^a^	22	17
Bull terrier^a^	12	7
Irish soft coated wheaten terrier^a^	2	0
Jack Russell terrier^a^	1	0
Wachtelhund^a^	1	0
Elkhound^b^	0	5
Hovawart^b^	0	8
Giant Schnauzer^b^	0	10
Smalands hound^b^	0	9
Irish wolfhound^b^	0	8
Mixed	0	1
Total number of dogs after quality controls	178	192

^a^ *HRCAD breeds,* ^b^ *LRCAD breeds*

### Two top-associated SNPs from the association analysis in GSDs

**Fig. 1:**
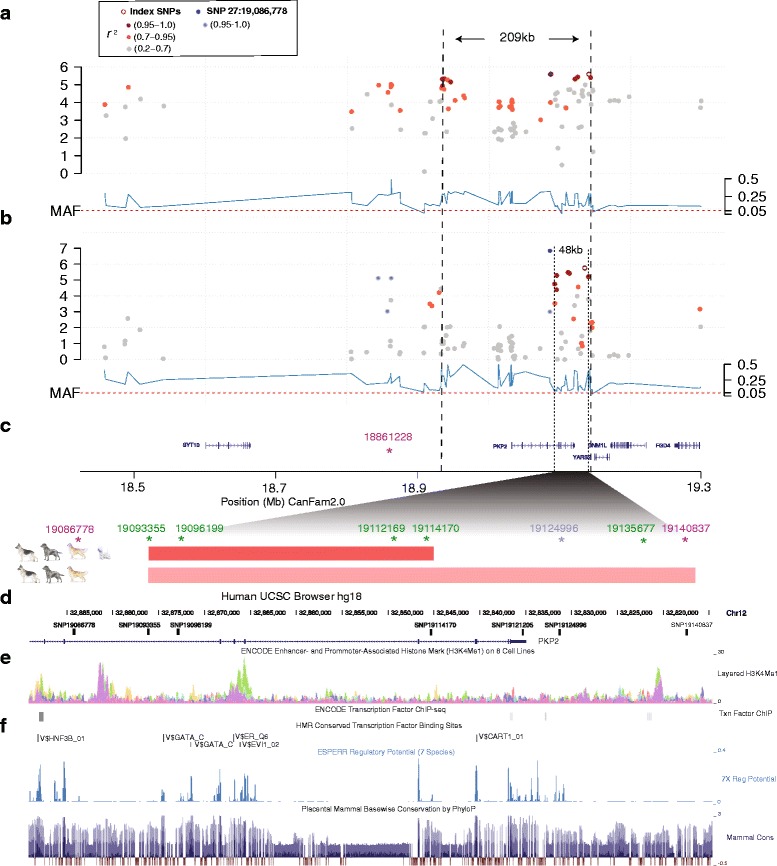
Association analyses in GSDs and LRCAD breeds revealed candidate variants located in tissue-specific enhancers. Association analyses including 104 fine-map SNPs located within the *PKP2* locus on CFA27 were performed separately in GSDs and in GSDs combined with LRCAD breeds. **a**. The GSD association analysis defined a ~209 kb associated region by associated SNPs displaying high linkage (r^2^ > 0.95) with the two top SNPs 27:19,086,778 (blue and index SNP) and 27:19,140,837 (index SNP). **b**. The combined association analysis including LRCAD breeds revealed the highly significant top SNP 27:19,086,778 (blue), and SNP 27: 19,135,677 (index SNP) in high LD (r^2^ > 0.95) with seven SNPs forming a ~48 kb risk haplotype. **c**. The ~48 kb risk haplotype (pink bar) stretched from *PKP2* intron 6 to the downstream region of the gene and was present in GSD, LR, and GR. A 21 kb risk haplotype (red bar) stretching from intron 6–10 was shared with WHWT. Asterisks and SNP positions mark the nine SNPs included in further analyses: purple and light blue SNPs were top SNPs in the GSD association and green SNPs were part of the ~48 kb risk haplotype. **d**. Positions of the SNPs in the human UCSC browser, where SNP 27:19,112,169 and 27:19,135,677 were not mapped from dog to human due to loss of conservation around the SNPs, and SNP 27: 27:19,124,996 (light blue) was not included in the luciferase experiments due to its location in a repetitive region. **e**. When matching the ~48 kb haplotype plus the SNP 27:19,086,778, to human ENCODE data, H3K4Me1 enhancer-associated histone modification marks were detected in normal human epidermal keratinocytes (NHEK) and human mammary epithelial cells (HMEC) (purple and green, respectively). **f**. The regulatory potential within the region is indicated by the tracks from the human UCSC browser (hg18): Transcription Factor ChIPSeq, HMR Conserved transcription factor binding sites, ESPERR and Regulatory Potential and conservation scores by PhyloP across placental mammals (https://genome.ucsc.edu/cgi-bin/hgGateway?db=hg18)

In a first attempt to narrow down the CAD-associated region in GSD, we performed association analysis in GSDs only (N_cases_ = 91 and N_controls_ = 83, same as in [20]) resulting in one additional top SNP 27:19,086,778 with the same p-value (p = 2.7 × 10^−6^) as the top GWAS SNP 27:19,140,837 [20]. These two SNPs were in very high linkage disequilibrium (LD; r^2^ ≥ 0.95) with eight other associated SNPs defining a ~209 kb region: from SNP 27:18,934,038 to SNP 27:19,143,309 (Fig. 1a and Additional file 3: Table S3).

### A ~48 kb risk haplotype in GSDs defined using additional breeds

While we were unable to narrow down the associated region using GSDs only, we hypothesized that other breeds could be used for breaking down the LD and thereby narrow down the CAD-associated region in GSDs. We therefore performed an association analysis in GSDs together with 5–10 control dogs from each of five different LRCAD breeds: Elkhound, Hovawart, Giant Schnauzer, Smalands hound, and Irish Wolfhound. CAD rarely affects these breeds and the sampled dogs were assumed to be CAD controls. This analysis was subsequently performed by comparing GSD CAD cases to CAD controls of GSD and five LRCAD breeds, and resulted in the same top SNP 27: 19,086,778 as in the GSD association analysis with a highly significant *p*-value (p = 1.4 × 10^−7^). The second most associated SNP 27:19,135,677 (p = 1.7 × 10^−6^) was in very strong LD (r^2^ ≥ 0.95, in the studied population) with six moderately associated SNPs (p = 3.3 × 10^−6^–4.1 × 10^−5^) defining a ~48 kb region covering half of the sixth intron to 19 kb downstream of *PKP2* (Fig. 1b and Additional file 4: Table S4). The risk alleles at these seven SNP positions formed a risk haplotype present in 36 % of the GSD cases and 10 % of the GSD controls and conferred an OR for CAD of 5.3 (95 % confidence interval, CI: 2.3–12.4, p = 3.1 × 10^−5^) in GSDs. For comparison, the more common risk allele at the top GWAS SNP 27:19,140,837 was present in 64 % of the cases and 31 % of the controls conferring with an OR of 3.9 (95 % CI: 2.1–7.2, Fisher’s exact probability test two-tailed: p = 2.1 × 10^−5^) for CAD. Next, we studied the genotypes of the dogs of HRCAD breeds listed in Table 1 and detected that the 48 kb risk haplotype was present at a high frequency (88 %) in LRs (in 16 out of 21 cases and in 12 out of 14 controls) and at a low frequency (4 %) in GRs (in one out of 10 cases and in none of the 15 controls). Half of the risk haplotype, consisting of five SNPs spanning ~21 kb from intron 6 to 10 of *PKP2*, was present in 58 % of the WHWTs: in 12 out of 18 cases (~42 kb in one WHWT case) and 7 out of 15 controls (Fig. 1c, Additional file 2: Table S2 and Additional file 5: Table S5). Due to the low sample numbers of these breeds, no conclusion could be drawn about association to CAD. The risk haplotype was not detected (nor part of it) in the other HRCAD breeds: Boxers (*N* = 39), Bull terriers (*N* = 19), Irish soft-coated wheaten terrier (*N* = 2), Jack Russell terrier (*N* = 1) or the dog of mixed breeds, or in any of the LRCAD breeds. Of note, the region including the ~48 kb shared haplotype and SNP 27:19,086,778 contains prominent H3K4Me1 enhancer-associated histone modification marks (based on ENCODE data) detected in normal human epidermal keratinocytes (NHEK) and human mammary epithelial cells (HMEC) (Fig. 1d).

### SNP 27:19,093,355 tags the ~48 kb risk haplotype

To evaluate all possible regulatory variants located in the ~48 kb risk haplotype, we considered the SNPs from the fine-mapping plus 107 additional SNPs observed in five previously re-sequenced GSDs [20]. Nine SNPs, in LD with the top two associated GWAS SNPs, were not detected in any of the LRCAD breeds, human, or other species, thus suggesting functional importance (see Materials and Methods and Additional file 6: Table S6). The best transcription factor (TF) binding site prediction (using TRAP [26, 27]) was detected for SNP 27:19,093,355 with the allele-specific binding of nine members of the GATA family of TFs with the most significant score for binding of GATA6 (*p* = 4.5 × 10^−4^) to the risk allele compared to the wild-type (p = 0.84; absolute difference log(p) = −3.3; Additional file 7: Table S7). SNP 27:19,093,355 was among the SNPs defining the 48 kb and the shorter 21 kb risk haplotype. We then genotyped additional dogs of the HRCAD breeds (GSDs, LRs, GRs, and WHWTs) for the SNP 27:19,093,355 and after quality controls, considering genotyping success and CAD status, the datasets were combined with the fine-map datasets. In LRs (N_cases_ = 130, N_controls_ = 110) and WHWTs (N_cases_ = 44, N_controls_ = 18) the frequencies of the risk allele were high in both cases and controls where 79 % of the LR cases and 82 % of the LR controls, and 55 % of the WHWT cases and 56 % of the WHWT controls carried the risk allele. In GRs (N_cases_ = 165, N_controls_ = 179) the risk allele was present in 3 % of the cases and in none of the controls. In the separate set of GSDs (N_cases_ = 73, N_controls_ =140), the risk allele was present in 29 % of the cases and 13 % of the controls conferring an OR of 2.7 (95 % CI: 1.3–5.6, p = 0.0054) for CAD. Since the risk allele of SNP 27:19,093,355 was unique to HRCAD breeds in our fine-map dataset, we evaluated its presence in other breeds by genotyping various number of dogs representing in total 43 different breeds (Additional file 8: Table S8). In addition to the GSD, GR, LR and WHWT, the risk allele was also present in the eight breeds: Bearded collie, Border collie, English springer spaniel, Finnish Lapphund, Giant Schnauzer, Nova Scotia duck tolling retriever, Poodle, and Welsh springer spaniel (Additional file 9: Table S9 and Additional file 10: Table S10).

### Nine SNPs define one major risk and one major protective haplotype in GSDs

For further validation, we selected the top SNPs from both GWAS [20] and fine-mapping analyses (Fig. 1c, Table 2 and Additional file 11: Table S11). These in total nine SNPs covered a ~280 kb region and we identified 21 different haplotypes (Additional file 12: Table S12) across all breeds listed in Table 1 (Additional file 2: Table S2). The most common haplotype in GSD (haplotype 5) consisted of the control alleles (i.e. alleles more frequent in control GSDs compared to case GSDs) at all nine SNP loci and was present in 11 breeds, i.e. all breeds except LR, WHWT, and Jack Russell terrier. In GSDs, haplotype 5 was present in 98 % of the controls and 85 % of the cases, and conferred a protective effect on CAD (OR = 0.14 with 95 % CI: 0.03–0.6; p = 0.0032). The risk alleles at all SNP loci defined another haplotype (haplotype 20) detected only in the HRCAD breeds GSD, LR, and GR. In GSDs, 35 % of the cases carried haplotype 20 compared to 10 % of the controls. Thus, this haplotype conferred a high risk for CAD (OR = 5.1 with 95 % CI: 2.2–11.9, *p* = 5.9 × 10^−5^) in GSDs (Table 2).

**Table 2:** Nine SNPs selected for functional evaluation

SNP	SNP 27:18,861,228	SNP 27:19,086,778	SNP 27:19,093,355	SNP 27:19,096,199	SNP 27:19,112,169	SNP 27:19,114,170	SNP 27:19,124,996	SNP 27:19,135,677	SNP 27:19,140,837	Haplotype ID	GSD cases (%) ^a^	GSD controls (%) ^a^	p-value^b^	OR
Control alleles	C	C	C	T	A	C	T	G	T	5	84.6	97.6	0.0032	0.14 (95 CI: 0.03–0.6)
Risk alleles	A	T	T	G	G	G	A	A	G	20	35.2	9.6	5.9×10^−5^	5.1 (95 CI: 2.2–11.9)
Association p-value in GSD	1.3×10^−5^	2.7×10^−6^	7.1×10^−5^	2.8×10^−5^	1.9×10^−5^	1.9×10^−5^	3.8×10^−6^	1.0×10^−5^	2.7×10^−6^					
Rank in association analyses:														
GWAS	2	na	na	na	na	na	na	na	1					
GSD fine-map	16	1	37	26	21	20	3	12	1					
GSD + LRCAD breeds	64	1	9	4	2	3	14	1	15					

Haplotype 5 was present in GSD, Boxer, Smalands hound, Golden retriever, Bull terrier, Elkhound, Hovawart, Giant Schnauzer, Irish wolfhound, Irish soft-coated wheaten terrier, and Wachtelhund

Haplotype 20 was present in GSD, Labrador and Golden retriever

^a^ Dogs either heterozygous or homozygous for the allele in %

^b^ Fisher’s Exact probability test, two-tailed

### Eight candidate SNPs located in cell type-specific enhancers

**Fig. 2:**
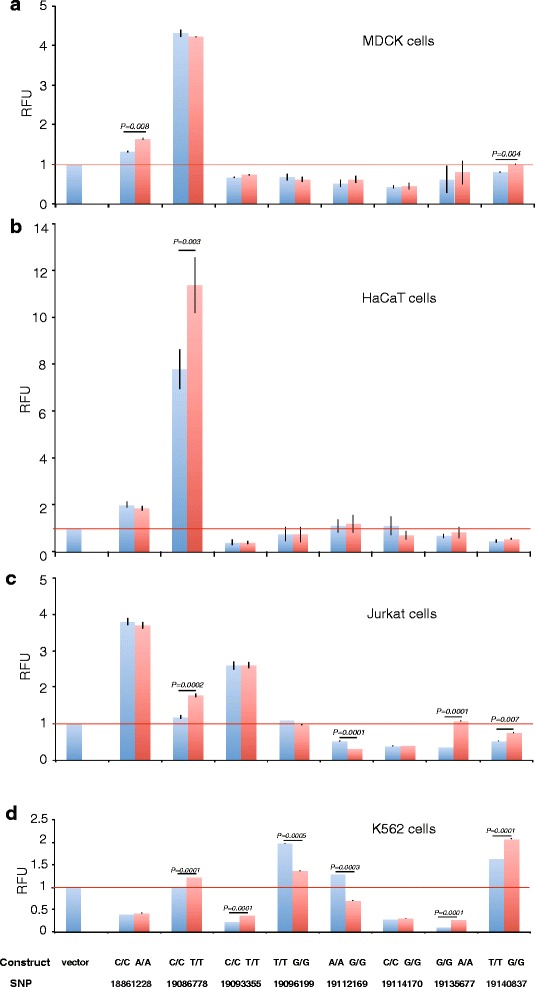
Regulatory potential and allelic differences of candidate regions. Luciferase reporter assays for each of the eight evaluated SNPs (SNP 27:18,861,228, SNP 27:19,086,778, SNP 27:19,093,355, SNP 27:19,096,199, SNP 27:19,112,169, SNP 27:19,114,170, SNP 27:19,135,677, and SNP 27:19,140,837) were performed in four different cell lines: **a**. MDCK, **b**. HaCaT, **c**. Jurkat, and **d**. K562. The cloned fragments showed differential activity in different cell lines, indicating the presence of cell type-specific enhancers. The fragment with the SNP 27:19,086,778 contained the most potent enhancer showing the highest activity in HaCaT cells (8 vs. 11-fold induction) with significant allelic difference (p = 0.003). All except SNP 27:19,114,170 showed allelic differences in at least one cell line

**Fig. 3:**
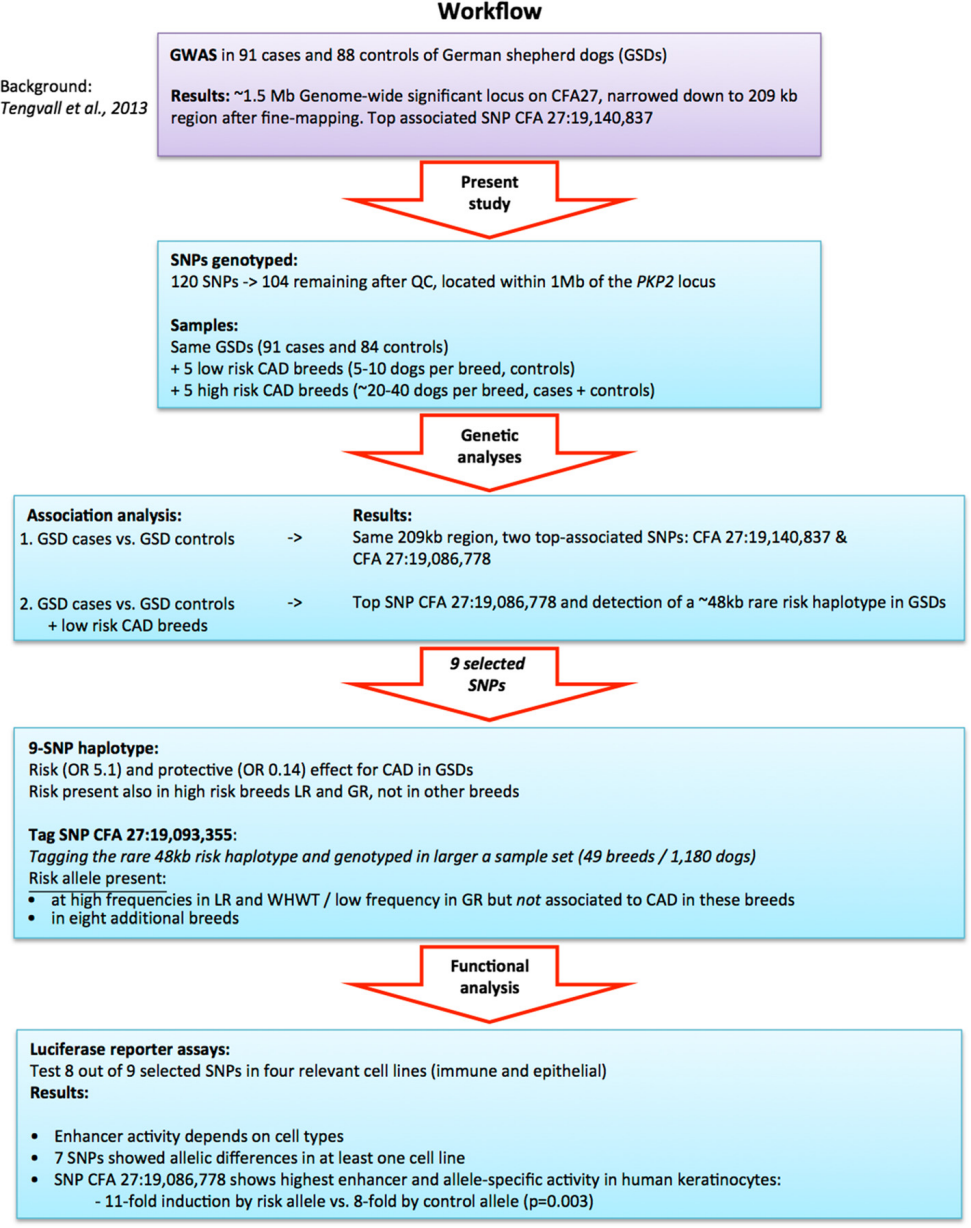
Summary of the workflow and major results. This study was initiated by the previous findings in [20], which thus serve as background (*purple text boxes*) for the present study (*light blue boxes*). Red arrows indicate the next step in the workflow

We used luciferase reporter assays to further evaluate the regulatory potential of the selected candidate variants. The SNP 27:19,124,996 was excluded due to the complicated repetitive region surrounding this SNP, leaving us with eight candidate variants for functional evaluation. Transient transfections followed by luciferase assays were performed using fragments ranging in size between 164 bp and 517 bp including the associated SNPs (Materials and Methods; Additional file 13: Table S13) in four different cell lines representing epithelial and immune cells that might be relevant to the disease: Madin-Darby canine epithelial cell line from Cocker spaniel (MDCK), human keratinocyte cell line (HaCaT), human T cell line (Jurkat), and human erythromyeloblastoid leukemia cell line (K562). We found that all eight regions have regulatory potential by either enhancing or inhibiting the activity of the luciferase gene, in at least one of the tested cell lines. Significant allele-specific differences on expression varied greatly between cell types and were detected for seven out of the eight SNPs (Fig. 2). An overview of the complete workflow starting from previous findings, through the current study design and the major results from both association analyses and functional analysis is presented in Fig. 3. The allelic differences on expression were more commonly observed in cells of hematopoietic origin (K562 and Jurkat), while the most profound effect on gene expression was detected for the SNP 27:19,086,778 in the epithelial cells (HaCaT and MDCK). Significant repressive effects by the risk allele compared to the control were detected for two regions (SNP 27:19,096,199 and SNP 27:19,112,169), whereas in the remaining six regions the risk allele always conferred an increased expression compared to the control allele when a significant allelic difference was observed.

The fragment containing SNP 27:18,861,228 showed the strongest effect on expression in Jurkat cells (4-fold induction compared to the control vector with a minimal promoter), but allelic difference was observed only in MDCK cells (*p* = 0.008). The fragment with the SNP 27:19,086,778 contained the most potent enhancer showing the highest activity in HaCaT cells (8 vs. 11-fold induction) with significant allelic difference (*p* = 0.003), whereas a 4-fold induction with no allelic difference in MDCK cells and allelic differences in Jurkat cells (*p* = 0.0002, 2-fold) and K562 (*p* = 0.0001) was observed. The fragment with the SNP 27:19,093,355 displayed an allelic difference (p = 0.0001) only in K562 cells consisting of repressor effects (<0.5-fold), whereas enhancer effects were detected in Jurkat cells (2.5-fold) and repressor effects in HaCaT cells (<0.5-fold) without allelic differences. The SNP 27:19,096,199-fragment showed a repressive effect by the risk allele compared to the control in K562 cells (*p* = 0.0005, 2-fold). Similarly, the SNP 27:19,112,169-fragment displayed repressive effects by the risk allele in K562 cells (*p* = 0.0003, <0.5-fold) and Jurkat cells (*p* = 0.0001, <0.5-fold). The SNP 27:19,114,170-fragment displayed repressive effects in MDCK, Jurkat, and K562 cells (<0.5-fold) without allelic differences. The SNP 27:19,135,677-fragment was acting as a repressor of gene transcription with allelic differences in Jurkat cells (<0.5-fold, *p* = 0.0001) and K562 cells (<0.2-fold, *p* = 0.0001). The SNP 27:19,140,837-fragment increased expression in K562 cells (2-fold) with an allelic difference (p = 0.0001), and allelic differences were also observed in MDCK cells (p = 0.004) and Jurkat cells (*p* = 0.007).

## Discussion

Whilst the structure of the dog genome makes dogs extremely useful for the initial mapping of complex disease genes, the presence of long regions with LD within purebred dogs may eventually hamper further genetic analysis of causative variants. Due to the extensive LD, we were unable to narrow down the CAD-associated haplotype in GSDs alone. Instead, we performed an across-breed analysis with a low number of control dogs of LRCAD breeds (N_breeds_ = 5, N_dogs_ = 40) together with 91 CAD cases and 84 controls of GSDs and defined one top-associated SNP 27:19,086,788 and a rare 48 kb risk haplotype in GSDs overlapping with regions of high regulatory potential within the *PKP2* gene. The low sample numbers of the LRCAD breeds and the large sample set of GSDs included in the analysis resulted in an association analysis still focused on CAD association in GSDs, with the exception that SNPs with risk alleles not present in the LRCAD breeds increased in significance. Whereas SNPs with risk alleles present in the LRCAD breeds were reduced in significance. One can argue that the low sample numbers of the LRCAD breeds may implicate that risk alleles by chance was not present in the particular dogs included in the analysis and also that other LRCAD breeds could possibly carry the risk alleles. The additional genotyping of SNP: 27:19,093,355 (tagging the 48 kb risk haplotype) did reveal that the risk allele at this locus was rare but not unique to defined HRCAD breeds nor was it the major segregating risk factor in LR, GR, or WHWT. Nevertheless, the association analysis resulted in the highly significant top SNP 27:19,086,778 and clearly distinguished the rare 48 kb risk haplotype with an OR for CAD higher than the top GWAS SNP in GSDs. When continuing the evaluation of the candidate SNPs defined by the association analyses, we observed allele-specific and cell type-specific differences of expression using reporter assays where some regions were more active in epithelial cells while others functioned in cells of hematopoietic origin.

Our findings presented here are in line with the “multiple enhancer variant” hypothesis stating that multiple SNPs in LD may influence multiple enhancers for a gene [28], each with a modest effect on gene expression in specific cell types. The very strong increase of the reporter expression by the fragment with the SNP 27:19,086,778 in the skin-relevant cell lines, but not in Jurkat and K562 representing two hematopoietic cell types, is in agreement with the lack of prominent signals for ENCODE enhancer-associated marks in hematopoietic cells within the locus. Moreover, the genomic region including the ~48 kb haplotype plus the flanking top GSD SNP 27:19,086,778, includes two strong ENCODE enhancer regions active in keratinocytes and epithelial cell lines (Fig. 1d). Overall, we detected rather modest enhancing and even repressive activities of the regulatory regions in the cell lines of hematopoietic origin, which may indicate that the locus is repressed in these cell types. Indeed, in an mRNA sequencing study, *PKP2* mRNA is barely detectable in dog blood [29], but the risk alleles for some of the SNPs might release this repression and activate the expression of the target genes in immune cells as well. The risk alleles for only two of the variants (SNP 27:19,096,199 and SNP 27:19,112,169) significantly reduce expression of the luciferase reporter in contrast to other associated SNPs, and this also suggest that they act independently of the other SNPs, or even on another gene. A recent report showed that enhancer regions in dogs may be separated from the affected gene by several non-relevant genes and more than 1 Mb [30]. Thus, the genes *DNM1L*, *YARS2,* or *FGD4* located close (~30-150 kb) to *PKP2,* may also be regulated by enhancers harboring the candidate variants despite their location within *PKP2*. Of note, the protein encoded by *FGD4* may be relevant for CAD development as it is expressed in hematopoietic cells, and has functions implicated in allergen-sensitized dendritic cells [31] and in parasite invasion through the gastro-intestinal tract [32].

A recent study in dogs showed that *PKP2* mRNA expression was significantly up-regulated in atopic skin compared to healthy skin. The difference was detected in non-lesional skin vs. control skin (p = 0.03) and was even more pronounced in lesional-skin vs. control skin (p = 0.001) [33]. The position of the candidate variants defined and evaluated in the present study were located close to the epithelial-specific enhancer region within the *PKP2* gene. Especially the fragment containing SNP 27:19,086,778 with strong enhancer effects on gene transcription supports the notion that the effect on CAD, by this locus, originates primarily from the *PKP2* gene and its regulation by this enhancer in the skin. Furthermore, the significantly higher expression of the fragment with the risk allele compared to the control allele in keratinocytes suggests that the SNP 27:19,086,778 risk allele contributes to an increased transcription of *PKP2* in the skin of CAD-affected GSDs.

To conclude if the expression of *PKP2* and/or any other genes in the nearby region is altered due to the risk variants, mRNA and protein expression analyses in tissues from relevant dogs are necessary. Relevant tissues may include both immune cells, and skin as well as additional epithelial tissues such as intestine epithelium as it is known that intestine integrity and allergen uptake is of high relevance in atopic skin manifestations [34, 35]. Skin tissue samples of lesional skin should optimally be collected from untreated CAD-cases to avoid interference by treatment on gene and protein expression, and healthy skin of control GSDs should be collected from the same body locations for comparison. Though, the exact effect on transcription caused by each variant as well as by the combination of variants may only be revealed using advanced experiments of live rodent models and gene-editing methods such as the CRISPR Cas9 technology [36].

CAD is a complex disease where genetic risk factors at multiple loci in combination with environmental risk factors contribute to CAD development. In GSDs, low serum IgA levels are highly correlated with CAD, thus this is one important risk factor for CAD defined in this breed [20, 37]. Also, interaction of genes within the *PKP2* locus and other genetic risk loci, including the newly identified loci associated with IgA levels in GSDs [38], may be of substantial importance for CAD development in GSDs. Even within the associated region on CFA27 (defined by GWAS) additional regulatory variants may be contributing to the CAD predisposition seen in GSDs. In our data set, only HRCAD breeds carried the 48 kb risk haplotype (GSD, LR, and GR) or part of it (WHWT). However, no association with CAD was detected in LRs, WHWTs, and GRs for the tagging SNP 27:19,093,355 genotyped in the extended datasets. The high frequencies of the risk allele in LRs (80 %) and WHWTs (55 %) may partly explain the overall increase of CAD in the breeds but where the main segregating risk factors are located within other breed-specific loci. The control haplotype 5, consisting of control alleles at the nine candidate SNP positions covering a region of 280 kb, conferred a protective effect in GSDs and was present in almost all breeds included in the fine-mapping. Interestingly, LR and WHWT (and Jack Russell Terriers: sample size = 1) were the only breeds in which the control haplotype 5 was not detected. Perhaps, this may also contribute to the lack of signal in LR and WHWT where the majority of dogs within these breeds carry regulatory risk variants across this locus.

## Conclusions

This study demonstrates that the contribution from the *PKP2* locus to CAD development in GSDs is dependent on several regulatory risk variants influencing cell type-specific transcription. This highlights the complexity of the associated locus and its effect on gene regulation, but also the complex nature of the disease involving systemic immune responses and skin damage. An altered expression pattern of the target gene(s) in either immune or epithelial cells may lead to perturbations in different signaling pathways, which jointly contribute to pathological changes in CAD. It is also possible that different enhancers actually regulate different genes located within this locus, and therefore we may expect even more complex tissue-specific changes in the expression pattern. No single causative variant could be identified, and we conclude that part of the predisposition to CAD in GSDs can be explained by multiple regulatory variants. These are located in tissue-specific enhancers within the *PKP2* locus that most likely jointly participate in transcriptional regulation at this locus influencing *PKP2* mRNA expression and nearby genes.

## Methods

### Sampling

Blood samples were collected from privately owned dogs in collaboration with several veterinary clinics throughout Sweden and Switzerland. Informed owner consent was obtained for each dog.

### DNA preparation

Genomic DNA was extracted from EDTA blood samples using the Qiagen mini- and/or midiprep extraction kit (Qiagen, Hilden, Germany). DNA samples were diluted in H_2_O and stored at −20 °C until used for genotyping.

### Phenotype classifications

Phenotype definitions of the CAD cases and controls are described in detail in [20]. In short, CAD cases had positive reactions on allergen-specific IgE tests, after carefully ruling out other causes of pruritus including a conducted diet trial to define cutaneous adverse food reactions. CAD controls were older than five years of age with no history of pruritus, repeated ear infections or skin lesions compatible with CAD. Based on clinical experience by veterinary dermatology specialist (author Kerstin Bergvall, KB), Giant Schnauzer, Hovawart, Irish wolfhound, Smalands hound, and Norwegian elkhound were defined as typical LRCAD breeds. Based on previous publications Boxer, Bull terrier, WHWT, GSD, LR, [8–11], GR [10, 12] and Soft-coated wheaten terrier, and Wachtelhund [8] were classified as HRCAD breeds.

### Selection of SNPs for genotyping

We used the targeted re-sequencing data generated using a 385 K custom-designed sequence capture array from Roche NimbleGen, WI, from five GSDs where in total 9503 SNPs were identified in the entire 2.8 Mb region on CFA27 using the canine genome version: CanFam2.0 (Additional file 14: Table S14) [20]. We used the same canine genome version (CanFam2.0) for assigning SNP positions throughout the current study to make comparisons to our previous study [20] feasible. The top-associated region within the *PKP2* locus covered ~209 kb (block 7–11), and due to the LD pattern (see Figure 4 in [20]) we included an extended candidate region of ~340 kb stretching from SNP 27:18,804,142 (r^2^ > 0.8 with neighboring SNP in block 7) to SNP 27:19,142,893 (end of block 11). Within this region, 894 SNPs followed the same pattern as the top two GWAS SNPs in the re-sequenced GSDs: one case homozygous for the risk allele (T6), two heterozygous cases (T7 and T8), and two controls homozygous for the control allele (T1 and T2; Additional file 15: Table S15).

In total, 102 SNPs within the 340 kb candidate region (selected SNPs are in the fourth column in Additional file 15: Table S15) were selected based on the following criteria: 1) 35 SNPs were selected based on the comparison of our set of SNPs to the SNPs identified in previously published whole genome sequence data from six dog- and wolf pools (described in [25]). We made our own division, based on clinical experience (KB), into dog pools with LRCAD breeds: Pool 2 (Smalands hound, Norwegian elkhound, Swedish elkhound, and Finnish Lapphund), Pool 4 (Drever) and Pool 5 (Belgian Tervueren). Pool 3 (English cocker spaniel, Springer spaniel, GR, and LR) and Pool 6 (Bearded collie, Hovawart, Giant schnauzer, and GSD) were considered as HRCAD pools based on the presence of the HRCAD breeds GR, LR, and GSD. Pool 1 (the wolf pool) was considered as either or, as we do not know if the CAD risk factor(s) arose before or after dog domestication. We compared SNPs from our GSDs to the SNPs identified with the pool data within the 340 kb region, and considered *i*) SNPs from the GSD data that were not found in the pool data (thus possibly functionally important) and *ii*) SNPs where LRCAD were fixed for the GSD control allele, as candidates. 2) 24 SNPs were located in the human regulatory region of *PKP2* (according to the UCSC human browser) corresponding to dog CFA27 ~ 19.01–19.04 Mb. 3) 36 SNPs were chosen in order to cover the whole 340 kb candidate region. 4) For comparison, we added the already genotyped top two GWAS SNPs, and the top six SNPs from the previous fine-mapping (SNP 27:19,140,837 was the top-associated SNP in both categories).

We also included 20 SNPs outside the defined 340 kb region by choosing eight SNPs within 18.45–18.54 Mb, six SNPs around 19.14–19.15 Mb, and six SNPs within 19.17–19.30 Mb (Additional file 16: Table S16). These regions showed association (yet, lower than the top region) in the previous fine-mapping (see details in [20]).

The final number of selected SNPs was 122, out of which 120 were successfully designed into four pools for genotyping using the iPLEX Sequenom MassARRAY platform.

### Association analyses in GSDs and other breeds

We used the GenABEL package ver. 1.8-0 [39], a part of R statistical suite/software, ver. 0.98.932 [40] for the quality control and association analyses. The simplest model qtscore (fast score test for association between a trait and genetic polymorphism) was applied. In total, 381 dogs and 120 SNPs were genotyped and 104 SNPs remained after quality controls (Additional file 1: Table S1) due to the exclusion of 16 SNPs with call rate < 0.1. The sample set consisted of the same GSDs used in the GWAS [20] (except for two GSD controls missing DNA) and CAD cases and controls of typical HRCAD breeds and LRCAD breeds. In total, 11 samples (three WHWTs, two Bull terriers, one Boxer, three GSDs, one Smalands Hound, and one Rhodesian ridgeback) were excluded due to call rate below 60 %. The 370 samples remaining for the analyses were 329 dogs of HRCAD breeds and 41 control dogs of LRCAD breeds (Table 1). Association analyses were performed in GSDs only and in GSDs combined with the CAD controls of LRCAD breeds (the mixed breed excluded). While the fine-mapped region was defined as genome-wide significant based on permutations in [20], we set out to select the top-associated SNPs within the region which would potentially have the strongest impact on gene regulation and thus with most effect on the phenotype.”

### Selection of candidate SNPs within the 48 kb associated haplotype

We used the targeted re-sequencing data from five GSDs [20] to systematically go through an extended version of the 48 kb associated haplotype that spanned ~52 kb from SNP 27:19,088,686 to SNP 27:19,140,837 (including the regions to the next genotyped SNP). From 114 SNPs called in this region, we selected candidates that were in LD with the top two GWAS SNPs in the GSDs re-sequence data, and with risk alleles not present in previously published whole genome sequence data from LRCAD breed pools [25], human, or other species (see Methods). We also excluded SNPs in repeat elements and those not mapped in the human genome. Using these criteria, nine candidate SNPs were selected, out of which six were included in the fine-map genotyping of 120 SNPs and five were among the 104 SNPs remaining after quality controls (Additional file 6: Table S6). The mutated and the wild-type sequences of the nine candidates were screened for transcription factor binding site predictions by the web-tool TRAP [26, 27] (http://trap.molgen.mpg.de/cgi-bin/trap_two_seq_form.cgi).

### Additional genotyping of SNP 27:19,093,355

Additional genotyping of SNP 27:19,093,355 was performed using TaqMan® (Lifetechnologies) protocol for allelic discrimination. We genotyped a few samples from a large number of breeds (N_dogs_ = 346, N_breeds_ = 43) and also a larger sample set of GR, LR, and WHWT, and GSD, obtained from other projects and collaborators (Additional file 8: Table S8).

### Odds ratio calculations

The ORs were calculated according to the following formula:

$$ OR=\frac{D_E/{H}_E}{D_{NE}/{H}_{NE}} $$

where D_E_ is the set of cases with the mutation (homozygous or heterozygous), H_E_ is the set of controls with the mutation, D_NE_ is the set of cases without the mutation (homozygous for the control allele), and H_NE_ is the set of controls without the mutation.

### Haplotype analysis

We selected the top SNPs from both analyses: SNP 27:19,086,778, and top second: SNP 27:19,140,837 and third: SNP 27:19,124,996 from the GSD association analysis as well as the top second GWAS SNP 27:18,861,228 [20]. Within the 48 kb risk haplotype (from the association of GSD and LRCAD breeds), we selected the four most associated SNPs: 27:19,135,677, 27:19,112,169, 27:19,114,170, 27:19,096,199, and the sixth most associated SNP 27:19,093,355 (based on the TF predictions). In total, nine SNPs were selected for further evaluation. We used PHASE 2.1.1 to define the nine-SNP haplotypes across all breeds and used Fisher’s Exact probability test (two-tailed) to examine the association of the haplotypes in GSDs.

### Luciferase reporter assays

The luciferase reporter assay was used to examine the regulatory potential of each of the following SNPs independently: SNP 27:18,861,228, SNP 27:19,086,778, SNP 27:19,093,355, SNP 27:19,096,199, SNP 27:19,112,169, SNP 27:19,114,170, SNP 27:19,135,677, and SNP 27:19,140,837. The corresponding dog genomic DNA fragments, harboring the SNPs, were PCR amplified and cloned in front of the minimal promoter in the pGL4.26 luciferase reporter vector (Promega). The sizes of the SNP-containing fragments used to construct the reporters for luciferase assays were as follows: SNP 27:18861228, 318 bp; SNP 27:19086778, 517 bp; SNP 27:19093355, 479 bp; SNP 27:19096199, 324 bp; SNP 27:19112169, 211 bp; SNP 27:19114170, 266 bp; SNP 27:19135677, 164 bp; SNP 27:19140837, 406 bp. The sequences of the fragments used are shown and the associated SNPs located in each fragment are indicated in bold red and additional SNPs in red (Additional file 13: Table S13). The plasmids were validated by sequencing and purified with EndoFree Plasmid Maxi Kit (Qiagen) for transfection into Madin Darby Canine Kidney epithelial cell line from Cocker spaniel (MDCK), human immortalized keratinocyte cell line (HaCaT), human T cell line (Jurkat), and human erythromyeloblastoid leukemia cell line (K562). All transfections were performed in the 24-well plates as follows: for Jurkat and K562: 7 × 105 cells/well were seeded in the RPMI-1640 medium supplemented with GlutaMAX and 10 % of heat-inactivated bovine serum just before the transfection; for MDCK and HaCaT: 3x10^5^ cells/well were seeded in the DMEM medium with GlutaMAX and serum 24 h prior to transfection. Lipofectamine 2000 (Invitrogen) was used in accordance with the manufacturer’s protocol to deliver DNA including 750 ng of the reporter plasmid and 50 ng of the pRL-TK (Promega) vector to cells in each well. Forty-eight hours after transfection, the cells were harvested and assayed for the Firefly and Renilla luciferase activities with the Dual-Luciferase Reporter Assay System (Promega). The luciferase activity provided by the control vector was set to 1, and then the test reporters’ activities as fold-change compared to the control. The experiment was repeated three times with four technical replicates for each plasmid and analyzed with a *t*-test.

## Abbreviations

AD, atopic dermatitis; CAD, canine atopic dermatitis; DNM1L, dynamin 1-like; FGD4, FYVE, RhoGEF and PH domain containing 4; GR, golden retriever; GSD, German shepherd dog; HaCaT, human keratinocyte cell line; HRCAD breeds, high-risk CAD breeds; Jurkat, human T cell line; K562, human erythromyeloblastoid leukemia cell line; LR, labrador retriever; LRCAD breeds, low-risk CAD breeds; MDCK, Madin Darby canine kidney epithelial cell line from Cocker spaniel; PKP2, plakophilin 2; WHWT, West Highland white terrier; YARS2, tyrosyl-tRNA synthetase

## Additional files

Additional file 1: Table S1. All SNPs included in the fine-mapping. (PDF 25 kb) Additional file 2: Table S2. Genotypes for 104 SNPs and haplotypes in all breeds. (TXT 161 kb) Additional file 3: Table S3. The complete results from association analysis in GSDs. (PDF 52 kb) Additional file 4: Table S4. The complete results from association analysis in GSD and LRCAD breeds. (PDF 53 kb) Additional file 5: Table S5. Phase results for seven SNPs in the 48 kb haplotype across all breeds. (PDF 28 kb) Additional file 6: Table S6. Selection criteria for SNPs within the targeted re-sequenced region of 52 kb at position CFA27: 19,088,686-19,140,837. (PDF 75 kb) Additional file 7: Table S7. SNP 27:19,093,355 prediction in sTRAP. (TXT 51 kb) Additional file 8: Table S8. Summary of dogs genotyped for SNP 27:19,093,355. (PDF 39 kb) Additional file 9: Table S9. Genotypes for candidate SNP 27:19,093,355 from the Taqman assay. (TXT 27 kb) Additional file 10: Table S10. Summary of additional breeds carrying the risk allele at SNP 27:19,093,355. (PDF 34 kb) Additional file 11: Table S11. Allele frequencies for all nine selected SNPs across all breeds. (PDF 50 kb) Additional file 12: Table S12. Phase results of nine SNPs across all breeds. (PDF 32 kb) Additional file 13: Table S13. DNA fragments and primers used for cloning with the luciferase reporter vector pGL4.26. (PDF 27 kb) Additional file 14: Table S14. All SNPs from targeted re-sequencing [20]. (TXT 250 kb) Additional file 15: Table S15. All SNPs in LD with the two top GWAS SNPs in GSDs within the candidate region: CFA27 18,804,142-19,142,893. (TXT 38 kb) Additional file 16: Table S16. Additional SNPs in LD with the two top GWAS SNPs in GSDs selected for genotyping in the 122 SNP set. (PDF 38 kb)
