# Supplementary material for: Multiple regulatory variants located in cell type-specific enhancers within the PKP2 locus form major risk and protective haplotypes for canine atopic dermatitis in German shepherd dogs
Source: BMC Genet. 2016 Jun 29;17:97. doi: 10.1186/s12863-016-0404-3 (PMC4928279; doi:10.1186/s12863-016-0404-3)
Supplement: Additional file 1: Table S1. — All SNPs included in the fine-mapping. (PDF 25 kb) [file 12863_2016_404_MOESM1_ESM.pdf]

Table S1. All SNPs included in the fine-mapping

SNP ID	Position in canfam2	Left after pooling	Left after QC	Position in canfam3
SNP 27:18457482	18457482	18457482	18457482	15448265
SNP 27:18459109	18459109	18459109	18459109	15449892
SNP 27:18459330	18459330	18459330	18459330	15450113
SNP 27:18486358	18486358	18486358	18486358	15477141
SNP 27:18486849	18486849	18486849	18486849	15477632
SNP 27:18490669	18490669	18490669	18490669	15481452
SNP 27:18507811	18507811	18507811	18507811	15498594
SNP 27:18540760	18540760	18540760	18540760	15531543
SNP 27:18805907	18805907	18805907	18805907	15796690
SNP 27:18806574	18806574	18806574	18806574	15797357
SNP 27:18808449	18808449	18808449	NA	15799232
SNP 27:18822997	18822997	18822997	NA	15813780
SNP 27:18827867	18827867	18827867	18827867	15818650
SNP 27:18844105	18844105	18844105	18844105	15834888
SNP 27:18857197	18857197	18857197	18857197	15846571
SNP 27:18861228	18861228	18861228	18861228	15850602
SNP 27:18861390	18861390	18861390	18861390	15850764
SNP 27:18862058	18862058	18862058	18862058	15851432
SNP 27:18862439	18862439	18862439	18862439	15851813
SNP 27:18870992	18870992	18870992	NA	15860366
SNP 27:18874358	18874358	18874358	18874358	15863732
SNP 27:18875109	18875109	18875109	18875109	15864483
SNP 27:18892590	18892590	NA	NA	15881964
SNP 27:18897701	18897701	18897701	NA	15887075
SNP 27:18907210	18907210	18907210	NA	15896584
SNP 27:18908562	18908562	18908562	18908562	15897936
SNP 27:18908958	18908958	18908958	18908958	15898332
SNP 27:18909840	18909840	18909840	18909840	15899214
SNP 27:18917097	18917097	18917097	18917097	15906471
SNP 27:18920067	18920067	18920067	18920067	15909441
SNP 27:18925907	18925907	18925907	NA	15915281
SNP 27:18930158	18930158	18930158	18930158	15919532
SNP 27:18932881	18932881	18932881	18932881	15922255
SNP 27:18932887	18932887	18932887	18932887	15922261
SNP 27:18934038	18934038	18934038	18934038	15923412
SNP 27:18934219	18934219	18934219	18934219	15923593
SNP 27:18934303	18934303	18934303	18934303	15923677
SNP 27:18936358	18936358	18936358	18936358	15925732
SNP 27:18936592	18936592	18936592	18936592	15925966
SNP 27:18940005	18940005	18940005	18940005	15929379
SNP 27:18941383	18941383	18941383	18941383	15930757

SNP 27:18942454	18942454	18942454	18942454	15931828
SNP 27:18945749	18945749	18945749	18945749	15935123
SNP 27:18951947	18951947	18951947	18951947	15941321
SNP 27:18964049	18964049	18964049	18964049	15953423
SNP 27:18965475	18965475	18965475	18965475	15954849
SNP 27:18982680	18982680	18982680	NA	15972054
SNP 27:19007642	19007642	19007642	19007642	15997016
SNP 27:19010982	19010982	NA	NA	16000356
SNP 27:19013243	19013243	19013243	19013243	16002617
SNP 27:19013318	**19013318**	19013318	19013318	16002692
SNP 27:19013715	19013715	19013715	19013715	16003089
SNP 27:19013764	19013764	19013764	19013764	16003138
SNP 27:19013850	19013850	19013850	19013850	16003224
SNP 27:19014281	19014281	19014281	19014281	16003655
SNP 27:19015640	19015640	19015640	19015640	16005014
SNP 27:19017040	19017040	19017040	NA	16006414
SNP 27:19017153	19017153	19017153	19017153	16006527
SNP 27:19017415	19017415	19017415	19017415	16006789
SNP 27:19028915	19028915	19028915	19028915	16018289
SNP 27:19030147	19030147	19030147	19030147	16019521
SNP 27:19031106	19031106	19031106	19031106	16020480
SNP 27:19031514	19031514	19031514	19031514	16020888
SNP 27:19031583	19031583	19031583	19031583	16020957
SNP 27:19031586	19031586	19031586	19031586	16020960
SNP 27:19031796	19031796	19031796	19031796	16021170
SNP 27:19031839	19031839	19031839	19031839	16021213
SNP 27:19032172	19032172	19032172	19032172	16021546
SNP 27:19032261	19032261	19032261	19032261	16021635
SNP 27:19032742	19032742	19032742	19032742	16022116
SNP 27:19032875	19032875	19032875	19032875	16022249
SNP 27:19033054	19033054	19033054	19033054	16022428
SNP 27:19034176	19034176	19034176	19034176	16023550
SNP 27:19034596	19034596	19034596	19034596	16023970
SNP 27:19036266	19036266	19036266	19036266	16025640
SNP 27:19037224	19037224	19037224	19037224	16026598
SNP 27:19052050	19052050	19052050	19052050	16041424
SNP 27:19052738	19052738	19052738	NA	16042112
SNP 27:19072862	19072862	19072862	19072862	16062236
SNP 27:19086631	19086631	19086631	19086631	16076005
SNP 27:19086778	19086778	19086778	19086778	16076152
SNP 27:19093355	19093355	19093355	19093355	16082729
SNP 27:19093585	19093585	19093585	19093585	16082959
SNP 27:19095978	19095978	19095978	19095978	16085352

SNP 27:19096199	19096199	19096199	19096199	16085573
SNP 27:19097058	19097058	19097058	NA	16086432
SNP 27:19097147	19097147	19097147	19097147	16086521
SNP 27:19099734	19099734	19099734	NA	16089108
SNP 27:19102681	19102681	19102681	19102681	16092055
SNP 27:19102841	19102841	19102841	19102841	16092215
SNP 27:19107516	19107516	19107516	19107516	16096890
SNP 27:19108790	19108790	19108790	19108790	16098164
SNP 27:19112169	19112169	19112169	19112169	16101543
SNP 27:19113531	19113531	19113531	NA	16102905
SNP 27:19114170	19114170	19114170	19114170	16103544
SNP 27:19119963	19119963	19119963	19119963	16109337
SNP 27:19121159	19121159	19121159	NA	16110533
SNP 27:19121205	19121205	19121205	19121205	16110579
SNP 27:19124996	19124996	19124996	19124996	16114370
SNP 27:19126533	19126533	19126533	19126533	16115907
SNP 27:19126638	19126638	19126638	19126638	16116012
SNP 27:19127748	19127748	19127748	NA	16117122
SNP 27:19128578	19128578	19128578	NA	16117952
SNP 27:19130125	19130125	19130125	19130125	16119499
SNP 27:19131113	19131113	19131113	19131113	16120487
SNP 27:19131614	19131614	19131614	19131614	16120988
SNP 27:19132339	19132339	19132339	19132339	16121713
SNP 27:19135677	19135677	19135677	19135677	16125051
SNP 27:19140837	19140837	19140837	19140837	16130211
SNP 27:19140960	19140960	19140960	19140960	16130334
SNP 27:19143309	19143309	19143309	19143309	16132683
SNP 27:19143986	19143986	19143986	19143986	16133360
SNP 27:19145949	19145949	19145949	NA	16135323
SNP 27:19146102	19146102	19146102	19146102	16135476
SNP 27:19146391	19146391	19146391	19146391	16135765
SNP 27:19146541	19146541	19146541	19146541	16135915
SNP 27:19170561	19170561	19170561	19170561	16159935
SNP 27:19177149	19177149	19177149	19177149	16166523
SNP 27:19182732	19182732	19182732	19182732	16172106
SNP 27:19197711	19197711	19197711	19197711	16187085
SNP 27:19298550	19298550	19298550	19298550	16287953
SNP 27:19299519	19299519	19299519	19299519	16288922
