# Supplementary material for: Multiple regulatory variants located in cell type-specific enhancers within the PKP2 locus form major risk and protective haplotypes for canine atopic dermatitis in German shepherd dogs
Source: BMC Genet. 2016 Jun 29;17:97. doi: 10.1186/s12863-016-0404-3 (PMC4928279; doi:10.1186/s12863-016-0404-3)
Supplement: Additional file 3: Table S3. — The complete results from association analysis in GSDs. (PDF 52 kb) [file 12863_2016_404_MOESM3_ESM.pdf]

Table S3. The complete results from association analysis in GSDs

Rank	SNP id	Chr	Position	LD pattern	A1	A2	N	effB	se_effB	chi2.1df	P1df	effAB	effBB
1	SNP 27:19086778	27	19086778	INDEX	C	T	169	3.529298	0.7522236	22.01317779	2.707849e-06	3.344444	13.0666667
2	SNP 27:19140837	27	19140837	INDEX	T	G	169	3.529298	0.7522236	22.01317779	2.707849e-06	3.344444	13.0666667
3	SNP 27:19124996	27	19124996	0.990716966225555	T	A	174	3.373878	0.7298436	21.36973424	3.787025e-06	3.094697	12.9545455
4	SNP 27:19143309	27	19143309	1	G	A	167	3.403088	0.7387958	21.21766562	4.099684e-06	3.188889	13.0666667
5	SNP 27:18936592	27	18936592	0.953165055644923	G	A	168	3.494494	0.7634230	20.95260762	4.707866e-06	3.361111	11.9166667
6	SNP 27:18934038	27	18934038	0.962606423748135	T	G	174	3.376690	0.7390469	20.87557220	4.901051e-06	3.166667	12.0909091
7	SNP 27:18934219	27	18934219	0.962606423748135	A	C	174	3.376690	0.7390469	20.87557220	4.901051e-06	3.166667	12.0909091
8	SNP 27:19121205	27	19121205	1	T	A	174	3.376690	0.7390469	20.87557220	4.901051e-06	3.166667	12.0909091
9	SNP 27:18941383	27	18941383	0.938035701925347	G	T	164	3.390142	0.7458157	20.66202344	5.479203e-06	3.281818	12.0909091
10	SNP 27:18945749	27	18945749	0.952192599581346	A	G	166	3.366801	0.7497934	20.16284309	7.112111e-06	3.208333	11.9166667
11	SNP 27:18861390	27	18861390	0.901078505888957	A	G	170	3.068275	0.6938186	19.55674673	9.765525e-06	3.113445	8.9411765
12	SNP 27:19135677	27	19135677	<0.8	G	A	174	6.909916	1.5666633	19.45333988	1.030874e-05	5.524554	Inf
13	SNP 27:18844105	27	18844105	0.943717710690249	T	G	174	3.242978	0.7372234	19.35038554	1.087973e-05	3.073529	10.8970588
14	SNP 27:18862439	27	18862439	0.943717710690249	C	T	174	3.242978	0.7372234	19.35038554	1.087973e-05	3.073529	10.8970588
15	SNP 27:18934303	27	18934303	0.952304105866912	G	C	171	3.203172	0.7309994	19.20111768	1.176445e-05	3.022727	11.2272727
16	SNP 27:18861228	27	18861228	0.943137209381987	C	A	173	3.186129	0.7316965	18.96114817	1.334075e-05	3.003676	10.8970588
17	SNP 27:18490669	27	18490669	0.943432273815804	A	G	174	3.116625	0.7181653	18.83302735	1.426750e-05	2.850000	11.4000000
18	SNP 27:18932887	27	18932887	0.828555211234474	G	A	174	4.891545	1.1326863	18.64974458	1.570683e-05	3.786713	Inf
19	SNP 27:18936358	27	18936358	0.934125139060208	G	A	174	3.115154	0.7272569	18.34777569	1.840337e-05	2.917857	10.5857143
20	SNP 27:19114170	27	19114170	<0.8	C	G	169	7.156344	1.6738667	18.27850778	1.908483e-05	5.481481	Inf
21	SNP 27:19112169	27	19112169	<0.8	A	G	174	7.157663	1.6752700	18.25462094	1.932565e-05	5.427632	Inf
22	SNP 27:19146102	27	19146102	<0.8	C	T	174	7.157663	1.6752700	18.25462094	1.932565e-05	5.427632	Inf
23	SNP 27:19131614	27	19131614	<0.8	T	A	171	6.384108	1.5032686	18.03545918	2.168284e-05	5.214286	Inf
24	SNP 27:19146391	27	19146391	<0.8	T	C	168	7.062653	1.6674278	17.94079411	2.278839e-05	5.407407	Inf
25	SNP 27:18857197	27	18857197	0.928202210673938	C	T	165	3.042545	0.7255809	17.58337587	2.749817e-05	2.869388	10.5857143
26	SNP 27:19096199	27	19096199	<0.8	T	G	171	6.813567	1.6272801	17.53171759	2.825546e-05	5.285714	Inf
27	SNP 27:19119963	27	19119963	<0.8	C	T	168	6.733922	1.6096107	17.50227178	2.869646e-05	5.310185	Inf
28	SNP 27:19093585	27	19093585	<0.8	A	G	167	6.646393	1.6037636	17.17478597	3.409316e-05	5.238426	Inf
29	SNP 27:19140960	27	19140960	<0.8	G	A	174	6.587750	1.5910248	17.14432457	3.464430e-05	5.172414	Inf
30	SNP 27:18827867	27	18827867	<0.8	G	A	169	6.574911	1.5884278	17.13344848	3.484325e-05	5.213636	Inf
31	SNP 27:19131113	27	19131113	<0.8	C	G	169	6.574911	1.5884278	17.13344848	3.484325e-05	5.213636	Inf

32	SNP 27:18964049	27	18964049	0.938055501480018	A	C	164	2.800562	0.6843602	16.74637308	4.272386e-05	3.363636	6.0454545
33	SNP 27:19132339	27	19132339	<0.8	G	A	166	7.410354	1.8197229	16.58316392	4.656254e-05	5.921053	Inf
34	SNP 27:19197711	27	19197711	<0.8	T	G	174	5.933317	1.4597927	16.52010486	4.813695e-05	4.760234	Inf
35	SNP 27:18965475	27	18965475	0.86584750510804	C	A	169	2.801983	0.6952953	16.24023264	5.579650e-05	3.042328	6.4814815
36	SNP 27:18507811	27	18507811	<0.8	C	T	165	7.894241	1.9780643	15.92721855	6.582527e-05	6.355932	Inf
37	SNP 27:19093355	27	19093355	<0.8	C	T	165	6.928052	1.7444089	15.77341137	7.139889e-05	5.701754	Inf
38	SNP 27:19032172	27	19032172	0.884318489679319	T	C	168	2.765653	0.6977120	15.71241801	7.373847e-05	2.981481	6.3518519
39	SNP 27:19177149	27	19177149	<0.8	T	C	169	5.091781	1.2880412	15.62715442	7.713886e-05	4.424242	Inf
40	SNP 27:18951947	27	18951947	0.844748508492122	T	C	172	2.773846	0.7021328	15.60724672	7.795526e-05	3.126437	5.7155172
41	SNP 27:18932881	27	18932881	<0.8	A	C	169	2.307496	0.5845830	15.58079090	7.905365e-05	4.562500	5.4315476
42	SNP 27:19170561	27	19170561	<0.8	T	C	170	5.599781	1.4221103	15.50513375	8.228148e-05	4.571429	Inf
43	SNP 27:19182732	27	19182732	<0.8	A	G	164	6.842695	1.7385835	15.49042404	8.292429e-05	5.625731	Inf
44	SNP 27:19299519	27	19299519	<0.8	A	G	164	6.842695	1.7385835	15.49042404	8.292429e-05	5.625731	Inf
45	SNP 27:19126533	27	19126533	<0.8	C	T	163	7.496198	1.9099847	15.40360202	8.682264e-05	6.206897	Inf
46	SNP 27:18930158	27	18930158	<0.8	A	G	173	5.756602	1.4707416	15.32003075	9.074914e-05	4.766949	Inf
47	SNP 27:18917097	27	18917097	<0.8	T	C	166	5.781645	1.4783713	15.29451960	9.198299e-05	4.811364	Inf
48	SNP 27:19013850	27	19013850	0.8738054705341	T	C	169	2.694756	0.6892664	15.28496861	9.244925e-05	2.869898	6.2500000
49	SNP 27:19014281	27	19014281	0.8738054705341	A	G	169	2.694756	0.6892664	15.28496861	9.244925e-05	2.869898	6.2500000
50	SNP 27:19031839	27	19031839	0.884590733168853	A	G	174	2.670868	0.6870453	15.11242131	1.012946e-04	2.853571	5.9500000
51	SNP 27:19032742	27	19032742	0.884590733168854	T	C	174	2.670868	0.6870453	15.11242131	1.012946e-04	2.853571	5.9500000
52	SNP 27:19086631	27	19086631	0.903233082706449	T	G	174	2.670868	0.6870453	15.11242131	1.012946e-04	2.853571	5.9500000
53	SNP 27:19143986	27	19143986	<0.8	G	A	160	7.224131	1.8907091	14.59895650	1.329882e-04	5.958621	Inf
54	SNP 27:18457482	27	18457482	0.906085657917015	T	C	174	2.616852	0.6850743	14.59093094	1.335558e-04	2.492308	7.2000000
55	SNP 27:18862058	27	18862058	<0.8	G	A	174	1.829149	0.4801588	14.51203452	1.392670e-04	NA	3.2930672
56	SNP 27:19095978	27	19095978	<0.8	A	G	169	5.970250	1.5774275	14.32472387	1.538312e-04	4.769737	NA
57	SNP 27:18540760	27	18540760	<0.8	C	T	166	5.351487	1.4196095	14.21055692	1.634509e-04	4.562500	Inf
58	SNP 27:19015640	27	19015640	0.861207842421221	G	C	167	2.603880	0.6917117	14.17070713	1.669495e-04	2.806122	5.8035714
59	SNP 27:19031586	27	19031586	0.872360432170529	A	G	164	2.570900	0.6831740	14.16145446	1.677725e-04	2.720000	5.9500000
60	SNP 27:19028915	27	19028915	0.882068338570704	C	A	171	2.567193	0.6850711	14.04254165	1.787212e-04	2.678571	5.8333333
61	SNP 27:18486358	27	18486358	<0.8	G	A	164	6.702220	1.7904783	14.01197047	1.816505e-04	5.750000	Inf
62	SNP 27:19107516	27	19107516	<0.8	T	C	166	4.576059	1.2273057	13.90201295	1.925921e-04	5.793651	1.3518519
63	SNP 27:19013715	27	19013715	0.854559215071244	G	A	174	2.502963	0.6722974	13.86072210	1.968702e-04	2.504464	5.9765625
64	SNP 27:19298550	27	19298550	<0.8	T	C	162	5.214901	1.4029619	13.81657393	2.015504e-04	4.500000	Inf

65	SNP 27:19108790	27	19108790	0.888583105001791	T	C	170	2.521142	0.6804807	13.72659890	2.114387e-04	2.620072	5.7580645
66	SNP 27:18942454	27	18942454	0.862564771125838	A	T	170	2.533159	0.6880896	13.55298691	2.319220e-04	2.625000	5.7166667
67	SNP 27:19032261	27	19032261	0.881785302962459	G	T	170	2.533159	0.6880896	13.55298691	2.319220e-04	2.625000	5.7166667
68	SNP 27:19032875	27	19032875	0.821075980770323	T	A	174	2.468316	0.6752965	13.36018152	2.570234e-04	2.578054	5.4558824
69	SNP 27:18874358	27	18874358	0.842270436897745	C	A	172	2.489376	0.6864761	13.15013486	2.874985e-04	2.644009	5.3467742
70	SNP 27:18805907	27	18805907	0.834706513829032	T	C	174	2.465768	0.6871934	12.87497818	3.330046e-04	2.618304	5.1796875
71	SNP 27:18459109	27	18459109	<0.8	T	C	174	4.136067	1.1980083	11.91943046	5.555146e-04	3.650000	Inf
72	SNP 27:18459330	27	18459330	<0.8	C	T	174	4.136067	1.1980083	11.91943046	5.555146e-04	3.650000	Inf
73	SNP 27:18920067	27	18920067	<0.8	G	A	172	3.082028	0.9214240	11.18804517	8.232603e-04	4.888393	1.9553571
74	SNP 27:19072862	27	19072862	0.833945166810764	T	G	174	2.171970	0.6580221	10.89498399	9.642502e-04	2.569412	3.8235294
75	SNP 27:19034176	27	19034176	<0.8	C	G	174	3.035473	0.9555356	10.09157739	1.489486e-03	3.390836	1.2830189
76	SNP 27:19036266	27	19036266	<0.8	G	A	174	2.882122	0.9473294	9.25598635	2.347269e-03	3.238095	1.2592593
77	SNP 27:19102841	27	19102841	<0.8	G	C	174	2.882122	0.9473294	9.25598635	2.347269e-03	3.238095	1.2592593
78	SNP 27:19130125	27	19130125	<0.8	A	C	174	2.882122	0.9473294	9.25598635	2.347269e-03	3.238095	1.2592593
79	SNP 27:19126638	27	19126638	<0.8	T	A	172	2.841468	0.9456117	9.02941944	2.656688e-03	3.207547	1.2830189
80	SNP 27:18806574	27	18806574	<0.8	G	A	174	2.716904	0.9144210	8.82787853	2.966631e-03	3.002597	2.4727273
81	SNP 27:19013318	27	19013318	<0.8	C	T	174	2.716904	0.9144210	8.82787853	2.966631e-03	3.002597	2.4727273
82	SNP 27:19030147	27	19030147	<0.8	A	G	173	2.789130	0.9436439	8.73617087	3.119589e-03	3.148148	1.2592593
83	SNP 27:19031583	27	19031583	<0.8	A	G	167	2.775306	0.9490063	8.55230995	3.450841e-03	3.142857	1.2941176
84	SNP 27:19031796	27	19031796	<0.8	C	T	167	2.775306	0.9490063	8.55230995	3.450841e-03	3.142857	1.2941176
85	SNP 27:18875109	27	18875109	<0.8	A	C	169	2.761828	0.9457332	8.52817494	3.496902e-03	3.129121	1.2884615
86	SNP 27:19013764	27	19013764	<0.8	C	A	169	2.761828	0.9457332	8.52817494	3.496902e-03	3.129121	1.2884615
87	SNP 27:19031106	27	19031106	<0.8	C	A	170	2.761619	0.9483613	8.47968624	3.591340e-03	3.113208	1.2452830
88	SNP 27:19031514	27	19031514	<0.8	G	A	170	2.761619	0.9483613	8.47968624	3.591340e-03	3.113208	1.2452830
89	SNP 27:19034596	27	19034596	<0.8	G	T	170	2.761619	0.9483613	8.47968624	3.591340e-03	3.113208	1.2452830
90	SNP 27:19017153	27	19017153	<0.8	A	G	174	2.738678	0.9420150	8.45213262	3.646156e-03	3.090909	1.2363636
91	SNP 27:18940005	27	18940005	<0.8	G	T	174	2.764479	0.9725626	8.07962793	4.476564e-03	3.179221	0.0000000
92	SNP 27:19052050	27	19052050	<0.8	T	A	166	2.683730	0.9468214	8.03417922	4.590281e-03	3.050420	1.2941176
93	SNP 27:19007642	27	19007642	<0.8	G	C	168	2.671097	0.9438260	8.00931740	4.653728e-03	3.037088	1.2884615
94	SNP 27:19037224	27	19037224	<0.8	T	C	167	2.633483	0.9459185	7.75092907	5.368492e-03	2.991758	1.2692308
95	SNP 27:18908958	27	18908958	<0.8	A	T	170	2.623291	0.9452691	7.70161776	5.517135e-03	2.968254	1.2222222
96	SNP 27:18909840	27	18909840	<0.8	A	T	170	2.623291	0.9452691	7.70161776	5.517135e-03	2.968254	1.2222222
97	SNP 27:19033054	27	19033054	<0.8	G	C	165	2.516784	0.9559437	6.93150026	8.469062e-03	2.840970	1.1698113

98	SNP 27:18486849	27	18486849	<0.8	A	G	174	2.019008	0.7934144	6.47553687	1.093693e-02	Inf	4.5021645
99	SNP 27:19013243	27	19013243	<0.8	A	C	164	2.506444	0.9933133	6.36712638	1.162537e-02	2.980519	1.2142857
100	SNP 27:19017415	27	19017415	<0.8	G	A	169	2.379402	0.9592351	6.15297874	1.311910e-02	2.748626	1.2321429
101	SNP 27:19097147	27	19097147	<0.8	A	G	172	1.751210	0.8458225	4.28664830	3.841282e-02	Inf	3.1184211
102	SNP 27:19146541	27	19146541	<0.8	A	G	173	3.400936	1.8181866	3.49880707	6.141305e-02	3.457317	Inf
103	SNP 27:19102681	27	19102681	<0.8	T	A	174	2.934321	3.0636403	0.91736010	3.381691e-01	NA	Inf
104	SNP 27:18908562	27	18908562	<0.8	C	T	174	1.191760	4.6004506	0.06710838	7.955945e-01	Inf	0.9213483
