# Supplementary material for: Multiple regulatory variants located in cell type-specific enhancers within the PKP2 locus form major risk and protective haplotypes for canine atopic dermatitis in German shepherd dogs
Source: BMC Genet. 2016 Jun 29;17:97. doi: 10.1186/s12863-016-0404-3 (PMC4928279; doi:10.1186/s12863-016-0404-3)
Supplement: Additional file 4: Table S4. — The complete results from association analysis in GSD and LRCAD breeds. (PDF 53 kb) [file 12863_2016_404_MOESM4_ESM.pdf]

**Table S4. The complete results from association analysis in GSD and LRCAD breeds**

| Rank | SNP ID | Chromosome | Position | LD pattern | A1 | A2 | N | effB | se_effB | chi2.1df | P1df | effAB | effBB |
|---|---|---|---|---|---|---|---|---|---|---|---|---|---|
| 1 | rs19086778 | 27 | 19086778 | <0.8 | C | T | 248 | 3.2381022 | 0.6157339 | 2.765636e+01 | 1.448952e-07 | 3.1965517 | 13.3189655 |
| 2 | rs19135677 | 27 | 19135677 | INDEX | G | A | 253 | 6.3535584 | 1.3285577 | 2.287037e+01 | 1.733027e-06 | 5.8318966 | Inf |
| 3 | rs19112169 | 27 | 19112169 | 0.972588001403266 | A | G | 253 | 6.6331224 | 1.4264927 | 2.162205e+01 | 3.320125e-06 | 5.7656250 | Inf |
| 4 | rs19114170 | 27 | 19114170 | 0.971944573425463 | C | G | 248 | 6.5373050 | 1.4147148 | 2.135304e+01 | 3.820146e-06 | 5.7411765 | Inf |
| 5 | rs19096199 | 27 | 19096199 | 0.985967968258574 | T | G | 250 | 6.3684561 | 1.3973085 | 2.077226e+01 | 5.172689e-06 | 5.6091954 | Inf |
| 6 | rs19140960 | 27 | 19140960 | 0.958963585433271 | G | A | 253 | 6.2545862 | 1.3833999 | 2.044098e+01 | 6.149878e-06 | 5.5280899 | Inf |
| 7 | rs18844105 | 27 | 18844105 | <0.8 | T | G | 253 | 2.7213639 | 0.6080370 | 2.003149e+01 | 7.617725e-06 | 2.5572650 | 10.2000000 |
| 8 | rs18862439 | 27 | 18862439 | <0.8 | C | T | 253 | 2.7213639 | 0.6080370 | 2.003149e+01 | 7.617725e-06 | 2.5572650 | 10.2000000 |
| 9 | rs19093355 | 27 | 19093355 | 0.966148638251957 | C | T | 244 | 6.5811311 | 1.5338205 | 1.840992e+01 | 1.781278e-05 | 6.0568182 | Inf |
| 10 | rs19126533 | 27 | 19126533 | 0.946403666067911 | C | T | 240 | 7.0126569 | 1.6724775 | 1.758104e+01 | 2.753195e-05 | 6.5258427 | Inf |
| 11 | rs18932881 | 27 | 18932881 | <0.8 | A | C | 248 | 2.1888329 | 0.5295090 | 1.708750e+01 | 3.569653e-05 | 2.7764706 | 5.1563025 |
| 12 | rs19095978 | 27 | 19095978 | 0.984653061224362 | A | G | 245 | 5.7945028 | 1.4122767 | 1.683421e+01 | 4.079126e-05 | 5.0416667 | NA |
| 13 | rs18930158 | 27 | 18930158 | 0.900866717691017 | A | G | 251 | 4.5253063 | 1.1304432 | 1.602502e+01 | 6.251102e-05 | 4.1797753 | Inf |
| 14 | rs19124996 | 27 | 19124996 | <0.8 | T | A | 253 | 1.9020943 | 0.4900191 | 1.506739e+01 | 1.037399e-04 | 3.0115830 | 3.1853282 |
| 15 | rs19140837 | 27 | 19140837 | <0.8 | G | T | 248 | 0.5262342 | 0.1394980 | 1.423056e+01 | 1.617228e-04 | 0.8807190 | 0.3145425 |
| 16 | rs18862058 | 27 | 18862058 | <0.8 | G | A | 253 | 1.6479388 | 0.4413714 | 1.394036e+01 | 1.887032e-04 | 0.0000000 | 2.8064516 |
| 17 | rs19093585 | 27 | 19093585 | 0.865785961976049 | A | G | 245 | 3.4474406 | 0.9528384 | 1.309047e+01 | 2.968025e-04 | 3.2375215 | Inf |
| 18 | rs18917097 | 27 | 18917097 | 0.899938581885164 | T | C | 244 | 3.6230130 | 1.0073105 | 1.293639e+01 | 3.222562e-04 | 3.4117647 | Inf |
| 19 | rs19121205 | 27 | 19121205 | <0.8 | A | T | 253 | 0.5557682 | 0.1570691 | 1.252004e+01 | 4.026099e-04 | 0.8238304 | 0.3376984 |
| 20 | rs18920067 | 27 | 18920067 | 0.815195567719666 | G | A | 250 | 2.8914782 | 0.8207218 | 1.241218e+01 | 4.265438e-04 | 4.2534884 | 2.0581395 |
| 21 | rs19298550 | 27 | 19298550 | 0.738817503230473 | T | C | 226 | 2.7834429 | 0.8184805 | 1.156506e+01 | 6.720265e-04 | 3.0518519 | 4.2916667 |
| 22 | rs18857197 | 27 | 18857197 | <0.8 | C | T | 243 | 2.0420108 | 0.6169945 | 1.095352e+01 | 9.342620e-04 | 1.7565657 | 6.1717172 |
| 23 | rs19086631 | 27 | 19086631 | <0.8 | T | G | 253 | 1.8778207 | 0.5705860 | 1.083094e+01 | 9.981802e-04 | 2.4483307 | 2.6352941 |
| 24 | rs18490669 | 27 | 18490669 | <0.8 | A | G | 253 | 1.6929178 | 0.5629020 | 9.044949e+00 | 2.634215e-03 | 1.7550000 | 2.8437500 |
| 25 | rs19119963 | 27 | 19119963 | 0.767892183209795 | C | T | 244 | 2.2912789 | 0.7670593 | 8.922736e+00 | 2.816422e-03 | 3.4786325 | 1.3703704 |
| 26 | rs19146391 | 27 | 19146391 | 0.856843519896086 | T | C | 247 | 2.3782681 | 0.8404354 | 8.007795e+00 | 4.657642e-03 | 3.3013575 | 0.6705882 |
| 27 | rs19143986 | 27 | 19143986 | 0.792363736388706 | G | A | 236 | 2.6097266 | 0.9291331 | 7.889221e+00 | 4.973028e-03 | 3.1395349 | 1.2790698 |
| 28 | rs18945749 | 27 | 18945749 | <0.8 | A | G | 242 | 1.6455647 | 0.6259914 | 6.910242e+00 | 8.570339e-03 | 1.9132653 | 2.3654917 |
| 29 | rs19015640 | 27 | 19015640 | <0.8 | G | C | 241 | 1.6404072 | 0.6243290 | 6.903612e+00 | 8.602179e-03 | 2.2649573 | 2.0512821 |
| 30 | rs19299519 | 27 | 19299519 | <0.8 | A | G | 242 | 1.9208861 | 0.7341973 | 6.845066e+00 | 8.888641e-03 | 2.6722689 | 1.7207792 |
| 31 | rs18908958 | 27 | 18908958 | <0.8 | A | T | 249 | 1.8008767 | 0.6894507 | 6.822782e+00 | 9.000226e-03 | 2.2787879 | 1.7090909 |

| | | | | | | | | | | | | | | |
|---|---|---|---|---|---|---|---|---|---|---|---|---|---|---|
| 32 | rs18909840 | 27 | 18909840 | <0.8 | A | T | 249 | 1.8008767 | 0.6894507 | 6.822782e+00 | 9.000226e-03 | 2.2787879 | 1.7090909 |
| 33 | rs18941383 | 27 | 18941383 | <0.8 | G | T | 241 | 1.6203284 | 0.6236099 | 6.751187e+00 | 9.368535e-03 | 1.9714286 | 2.2500000 |
| 34 | rs19146102 | 27 | 19146102 | 0.837861755206668 | C | T | 253 | 2.1513796 | 0.8352893 | 6.633768e+00 | 1.000634e-02 | 2.8500000 | 0.6477273 |
| 35 | rs18507811 | 27 | 18507811 | <0.8 | C | T | 244 | 1.6852099 | 0.6841988 | 6.066572e+00 | 1.377640e-02 | 2.6428571 | 1.6071429 |
| 36 | rs19143309 | 27 | 19143309 | <0.8 | A | G | 243 | 0.6793494 | 0.2829080 | 5.766285e+00 | 1.633653e-02 | 1.4745098 | 0.5067873 |
| 37 | rs19102681 | 27 | 19102681 | <0.8 | T | A | 253 | 0.5358962 | 0.2335411 | 5.265438e+00 | 2.175294e-02 | 0.1739812 | 0.4252874 |
| 38 | rs18940005 | 27 | 18940005 | 0.64100718017262 | G | T | 253 | 1.7731911 | 0.8228595 | 4.643653e+00 | 3.116860e-02 | 2.3793490 | 0.4362140 |
| 39 | rs19032875 | 27 | 19032875 | <0.8 | T | A | 252 | 1.4672010 | 0.6912065 | 4.505714e+00 | 3.378179e-02 | 1.8125000 | 1.8750000 |
| 40 | rs19131614 | 27 | 19131614 | 0.699123465865834 | T | A | 250 | 1.6990701 | 0.8014669 | 4.494190e+00 | 3.401022e-02 | 3.3777778 | 0.4444444 |
| 41 | rs19032742 | 27 | 19032742 | <0.8 | T | C | 253 | 1.4646158 | 0.6912811 | 4.488880e+00 | 3.411602e-02 | 2.0244898 | 1.7777778 |
| 42 | rs19028915 | 27 | 19028915 | <0.8 | C | A | 250 | 1.4375109 | 0.7143340 | 4.049670e+00 | 4.417997e-02 | 1.9285714 | 1.7500000 |
| 43 | rs18934303 | 27 | 18934303 | <0.8 | G | C | 249 | 1.3834166 | 0.7503552 | 3.399165e+00 | 6.522945e-02 | 1.8218623 | 1.6119910 |
| 44 | rs18486849 | 27 | 18486849 | <0.8 | A | G | 253 | 1.3512007 | 0.7394189 | 3.339325e+00 | 6.764237e-02 | Inf | 1.8031915 |
| 45 | rs18861390 | 27 | 18861390 | <0.8 | A | G | 249 | 1.3845377 | 0.7613895 | 3.306708e+00 | 6.899760e-02 | 1.5042735 | 1.8095238 |
| 46 | rs19030147 | 27 | 19030147 | 0.615493186043567 | A | G | 250 | 1.5302088 | 0.8560551 | 3.195199e+00 | 7.385476e-02 | 2.9145591 | 0.3858998 |
| 47 | rs18936592 | 27 | 18936592 | <0.8 | G | A | 245 | 1.3777168 | 0.7744772 | 3.164482e+00 | 7.525630e-02 | 2.0107411 | 1.4693878 |
| 48 | rs19108790 | 27 | 19108790 | <0.8 | T | C | 248 | 1.3490986 | 0.7629817 | 3.126505e+00 | 7.702872e-02 | 2.3845161 | 1.4000000 |
| 49 | rs19031583 | 27 | 19031583 | <0.8 | A | G | 246 | 1.5181761 | 0.8640107 | 3.087494e+00 | 7.889624e-02 | 2.8256757 | 0.3938224 |
| 50 | rs19031796 | 27 | 19031796 | <0.8 | C | T | 246 | 1.5181761 | 0.8640107 | 3.087494e+00 | 7.889624e-02 | 2.8256757 | 0.3938224 |
| 51 | rs19031106 | 27 | 19031106 | 0.616331752855009 | C | A | 249 | 1.5148389 | 0.8643764 | 3.071334e+00 | 7.968404e-02 | 2.8184211 | 0.3834586 |
| 52 | rs19031514 | 27 | 19031514 | 0.616331752855009 | G | A | 249 | 1.5148389 | 0.8643764 | 3.071334e+00 | 7.968404e-02 | 2.8184211 | 0.3834586 |
| 53 | rs19131113 | 27 | 19131113 | 0.738975315955175 | C | G | 248 | 1.5483067 | 0.9243774 | 2.805533e+00 | 9.393962e-02 | 3.3543956 | 0.2202381 |
| 54 | rs18934219 | 27 | 18934219 | <0.8 | A | C | 252 | 1.3017054 | 0.7880236 | 2.728645e+00 | 9.856289e-02 | 2.8070175 | 1.4800638 |
| 55 | rs18965475 | 27 | 18965475 | <0.8 | A | C | 247 | 0.7718637 | 0.4672946 | 2.728348e+00 | 9.858120e-02 | 1.6071429 | 0.5769231 |
| 56 | rs19034176 | 27 | 19034176 | <0.8 | C | G | 252 | 1.4241726 | 0.8661560 | 2.703541e+00 | 1.001256e-01 | 2.7659933 | 0.4227273 |
| 57 | rs18934038 | 27 | 18934038 | <0.8 | T | G | 253 | 1.2990174 | 0.7922076 | 2.688759e+00 | 1.010585e-01 | 2.7244582 | 1.4800638 |
| 58 | rs18936358 | 27 | 18936358 | <0.8 | G | A | 253 | 1.3276386 | 0.8156668 | 2.649318e+00 | 1.035943e-01 | 1.7909357 | 1.4660494 |
| 59 | rs18486358 | 27 | 18486358 | <0.8 | G | A | 226 | 1.4141382 | 0.8775915 | 2.596563e+00 | 1.070957e-01 | 2.2732198 | 1.1633987 |
| 60 | rs19014281 | 27 | 19014281 | <0.8 | G | A | 247 | 0.7811625 | 0.4996918 | 2.443871e+00 | 1.179847e-01 | 1.4400000 | 0.6057692 |
| 61 | rs19017153 | 27 | 19017153 | <0.8 | A | G | 253 | 1.4243950 | 0.9442996 | 2.275314e+00 | 1.314485e-01 | 2.4965035 | 0.3736264 |
| 62 | rs19013850 | 27 | 19013850 | <0.8 | C | T | 247 | 0.7933673 | 0.5332245 | 2.213749e+00 | 1.367859e-01 | 1.6605223 | 0.6108059 |
| 63 | rs18964049 | 27 | 18964049 | <0.8 | C | A | 243 | 0.8022793 | 0.5461377 | 2.157977e+00 | 1.418313e-01 | 1.9837662 | 0.6448749 |
| 64 | rs18861228 | 27 | 18861228 | <0.8 | C | A | 252 | 1.3032632 | 0.8968994 | 2.111430e+00 | 1.462026e-01 | 1.5037594 | 1.5037594 |

| | | | | | | | | | | | | | |
|---|---|---|---|---|---|---|---|---|---|---|---|---|---|
| 65 | rs19132339 | 27 | 19132339 | 0.719898138641465 | G | A | 245 | 1.4760137 | 1.0160374 | 2.110383e+00 | 1.463027e-01 | 3.4334038 | 0.2170543 |
| 66 | rs19032172 | 27 | 19032172 | <0.8 | C | T | 246 | 0.7918806 | 0.5527223 | 2.052605e+00 | 1.519460e-01 | 1.5834586 | 0.6336134 |
| 67 | rs18457482 | 27 | 18457482 | <0.8 | T | C | 253 | 1.2560209 | 0.8938761 | 1.974418e+00 | 1.599797e-01 | 2.1835687 | 1.3829268 |
| 68 | rs19033054 | 27 | 19033054 | 0.616003367079338 | G | C | 241 | 1.3915331 | 1.0072521 | 1.908581e+00 | 1.671209e-01 | 2.5263158 | 0.3609023 |
| 69 | rs19031586 | 27 | 19031586 | <0.8 | G | A | 241 | 0.8102734 | 0.6176767 | 1.720841e+00 | 1.895848e-01 | 1.5731092 | 0.6727763 |
| 70 | rs19102841 | 27 | 19102841 | 0.671869157865555 | G | C | 253 | 1.3690241 | 1.0643976 | 1.654301e+00 | 1.983746e-01 | 2.6961178 | 0.1824441 |
| 71 | rs19007642 | 27 | 19007642 | 0.627688820677887 | G | C | 246 | 1.3710637 | 1.0673436 | 1.650087e+00 | 1.989471e-01 | 2.7060478 | 0.1880651 |
| 72 | rs19072862 | 27 | 19072862 | <0.8 | T | G | 253 | 1.2427126 | 0.9848366 | 1.592257e+00 | 2.070040e-01 | 1.6358464 | 1.3157895 |
| 73 | rs18951947 | 27 | 18951947 | <0.8 | T | C | 248 | 1.2510492 | 0.9941441 | 1.583617e+00 | 2.082405e-01 | 1.9522574 | 1.1936170 |
| 74 | rs18942454 | 27 | 18942454 | <0.8 | T | A | 249 | 0.8257229 | 0.6662943 | 1.535806e+00 | 2.152431e-01 | 1.4935065 | 0.6722689 |
| 75 | rs19031839 | 27 | 19031839 | <0.8 | G | A | 253 | 0.8206863 | 0.6623230 | 1.535376e+00 | 2.153074e-01 | 1.6541353 | 0.6900270 |
| 76 | rs19126638 | 27 | 19126638 | 0.666610266420319 | T | A | 251 | 1.3540497 | 1.0943729 | 1.530871e+00 | 2.159818e-01 | 2.6571816 | 0.1846690 |
| 77 | rs19013715 | 27 | 19013715 | <0.8 | A | G | 253 | 0.8312201 | 0.6825809 | 1.482941e+00 | 2.233150e-01 | 1.6219055 | 0.6805616 |
| 78 | rs19013318 | 27 | 19013318 | 0.637404246285735 | C | T | 253 | 1.3167649 | 1.1467213 | 1.318563e+00 | 2.508502e-01 | 2.3684211 | 0.3571429 |
| 79 | rs19032261 | 27 | 19032261 | <0.8 | T | G | 249 | 0.8394065 | 0.7684211 | 1.193290e+00 | 2.746669e-01 | 1.5939850 | 0.7170868 |
| 80 | rs19017415 | 27 | 19017415 | <0.8 | G | A | 247 | 1.2907079 | 1.2072063 | 1.143123e+00 | 2.849934e-01 | 2.2133816 | 0.3688969 |
| 81 | rs18805907 | 27 | 18805907 | <0.8 | T | C | 253 | 1.1787112 | 1.2188848 | 9.351677e-01 | 3.335237e-01 | 1.9215071 | 1.0986395 |
| 82 | rs18827867 | 27 | 18827867 | <0.8 | G | A | 248 | 1.2346786 | 1.3147689 | 8.818790e-01 | 3.476875e-01 | 1.9494494 | 0.4814189 |
| 83 | rs18875109 | 27 | 18875109 | 0.618944943511058 | A | C | 248 | 1.2424185 | 1.3688360 | 8.238211e-01 | 3.640653e-01 | 2.5072368 | 0.1609375 |
| 84 | rs18908562 | 27 | 18908562 | <0.8 | C | T | 253 | 1.2763786 | 1.4710567 | 7.528357e-01 | 3.855799e-01 | 1.9587156 | 1.3431193 |
| 85 | rs19130125 | 27 | 19130125 | <0.8 | A | C | 253 | 1.2017513 | 1.4993419 | 6.424330e-01 | 4.228311e-01 | 2.4165708 | 0.1406470 |
| 86 | rs18932887 | 27 | 18932887 | <0.8 | G | A | 253 | 1.1411408 | 1.4762434 | 5.975339e-01 | 4.395205e-01 | 3.1812865 | 0.8692699 |
| 87 | rs18874358 | 27 | 18874358 | <0.8 | C | A | 250 | 1.1490651 | 1.5178000 | 5.731392e-01 | 4.490142e-01 | 1.4218182 | 1.0977273 |
| 88 | rs19177149 | 27 | 19177149 | <0.8 | T | C | 247 | 1.1551856 | 1.6919021 | 4.661795e-01 | 4.947501e-01 | 1.5338047 | 0.8127854 |
| 89 | rs19197711 | 27 | 19197711 | <0.8 | T | G | 253 | 1.1350774 | 1.9000145 | 3.568923e-01 | 5.502373e-01 | 1.5750000 | 0.6666667 |
| 90 | rs19146541 | 27 | 19146541 | <0.8 | A | G | 249 | 1.2793501 | 2.2708509 | 3.173961e-01 | 5.731767e-01 | 2.5833333 | 0.5535714 |
| 91 | rs19037224 | 27 | 19037224 | <0.8 | T | C | 239 | 0.9179664 | 1.7491827 | 2.754122e-01 | 5.997241e-01 | 2.1964286 | 0.6347403 |
| 92 | rs19182732 | 27 | 19182732 | <0.8 | A | G | 240 | 1.1186907 | 2.1756768 | 2.643816e-01 | 6.071258e-01 | 1.6153846 | 0.6666667 |
| 93 | rs19170561 | 27 | 19170561 | <0.8 | T | C | 249 | 1.1088726 | 2.2609311 | 2.405407e-01 | 6.238158e-01 | 1.6436937 | 0.6013514 |
| 94 | rs19034596 | 27 | 19034596 | <0.8 | G | T | 249 | 1.0814618 | 2.5397116 | 1.813233e-01 | 6.702385e-01 | 1.7134328 | 0.7919227 |
| 95 | rs19013243 | 27 | 19013243 | <0.8 | A | C | 239 | 1.0949399 | 3.0918981 | 1.254094e-01 | 7.232400e-01 | 2.1000000 | 0.1714286 |
| 96 | rs19052050 | 27 | 19052050 | <0.8 | A | T | 243 | 1.0532255 | 3.0699908 | 1.176979e-01 | 7.315449e-01 | 4.2656250 | 1.3442029 |
| 97 | rs18459109 | 27 | 18459109 | <0.8 | T | C | 253 | 0.9510056 | 3.3390027 | 8.112088e-02 | 7.757841e-01 | 2.0689855 | 0.5229994 |

| | | | | | | | | | | | | | |
|---|---|---|---|---|---|---|---|---|---|---|---|---|---|
| 98 | rs18459330 | 27 | 18459330 | <0.8 | C | T | 253 | 0.9510056 | 3.3390027 | 8.112088e-02 | 7.757841e-01 | 2.0689855 | 0.5229994 |
| 99 | rs18540760 | 27 | 18540760 | <0.8 | C | T | 241 | 1.0275796 | 6.8368148 | 2.259037e-02 | 8.805272e-01 | 2.3216783 | 0.6080586 |
| 100 | rs19036266 | 27 | 19036266 | <0.8 | G | A | 250 | 0.9826830 | 8.8879265 | 1.222436e-02 | 9.119622e-01 | 2.4164760 | 0.7430341 |
| 101 | rs18806574 | 27 | 18806574 | <0.8 | A | G | 253 | 0.9836671 | 9.2712114 | 1.125702e-02 | 9.155037e-01 | 2.5253623 | 1.1745086 |
| 102 | rs19097147 | 27 | 19097147 | <0.8 | A | G | 245 | 0.9921039 | 23.1030073 | 1.844069e-03 | 9.657473e-01 | Inf | 0.9592959 |
| 103 | rs19013764 | 27 | 19013764 | <0.8 | C | A | 247 | 0.9945397 | 34.7293195 | 8.200715e-04 | 9.771542e-01 | 1.7580492 | 0.5082721 |
| 104 | rs19107516 | 27 | 19107516 | <0.8 | T | C | 239 | 1.0011236 | 188.5490883 | 2.819204e-05 | 9.957636e-01 | 2.6571429 | 0.3428571 |
