# Supplementary material for: Multiple regulatory variants located in cell type-specific enhancers within the PKP2 locus form major risk and protective haplotypes for canine atopic dermatitis in German shepherd dogs
Source: BMC Genet. 2016 Jun 29;17:97. doi: 10.1186/s12863-016-0404-3 (PMC4928279; doi:10.1186/s12863-016-0404-3)
Supplement: Additional file 5: Table S5. — Phase results for seven SNPs in the 48 kb haplotype across all breeds. (PDF 28 kb) [file 12863_2016_404_MOESM5_ESM.pdf]

Table S5.Phase results of seven SNPs in the 48 kb haplotype across all breeds.

haplotypeID	haplotype	19093355	19095978	19096199	19112169	19114170	19135677	19140960	No of alleles
1	CATACGG	C	A	T	A	C	G	G	628
2	CATACAG	C	A	T	A	C	A	G	3
3	CATAGGG	C	A	T	A	G	G	G	1
4	TGGGGGG	T	G	G	G	G	G	G	21
5	TGGGGAG	T	G	G	G	G	A	G	3
6	TGGGGAA	T	G	G	G	G	A	A	84
