# Supplementary material for: Multiple regulatory variants located in cell type-specific enhancers within the PKP2 locus form major risk and protective haplotypes for canine atopic dermatitis in German shepherd dogs
Source: BMC Genet. 2016 Jun 29;17:97. doi: 10.1186/s12863-016-0404-3 (PMC4928279; doi:10.1186/s12863-016-0404-3)
Supplement: Additional file 6: Table S6. — Selection criteria for SNPs within the targeted re-sequenced region of 52 kb at position CFA27: 19,088,686-19,140,837. (PDF 75 kb) [file 12863_2016_404_MOESM6_ESM.pdf]

Table S6. Selection criteria for SNPs within the targeted re-sequenced region of 52kb at position CFA27: 19,088,686-19,140,837

chromosome	position	ref	alt	perfect pattern (ref=ctrl, T7/8=het)	genotyped in finemapping (120SNPs)	Allele freq in wolf	Allele freq in LRCADbreeds pool 2	conclusion after pattern and pool info	check cons in human etc.	Conclusion after comparing to human (mouse/rat)
chr27	19088686	G		yes		#N/A	#N/A	interesting- not variable in pools and perfect pattern	human(= A) = GSD CAD cases	human = A = cases
chr27	19089051	A				#N/A	#N/A	exclude - ref=case		
chr27	19089380	A				#N/A	#N/A	exclude - ref=case		
chr27	19089551	T				#N/A	#N/A	exclude - ref=case		
chr27	19089607	A				#N/A	#N/A	exclude - ref=case		
chr27	19089961	T				#N/A	#N/A	exclude - ref=case		
chr27	19090836	G		yes		#N/A	#N/A	interesting- not variable in pools and perfect pattern	human = G = controls	Interesting
chr27	19090841	C		yes		#N/A	#N/A	interesting- not variable in pools and perfect pattern	human = C = controls	Interesting
chr27	19092671	T				#N/A	#N/A	exclude - ref=case		
chr27	19092917	T				#N/A	#N/A	exclude - ref=case		
chr27	19093103	G		yes		#N/A	#N/A	interesting- not variable in pools and perfect pattern	not mapped in human	not mapped in human
chr27	19093355	C		yes	yes	0,571428571	0	interesting - pools match! (pool3 and wolf variable, rest is ref)	human = C = controls	Interesting
chr27	19093585	A		yes	yes	0,285714286	0	interesting - pools match! (pool3 and wolf variable, rest is ref)	human = A = controls	Interesting
chr27	19093812	T				#N/A	#N/A	exclude - ref=case		
chr27	19093824	A				#N/A	#N/A	exclude - ref=case		
chr27	19094167	A		yes		0,25	0,111111111	exclude - variation in pools		
chr27	19094520	G				#N/A	#N/A	exclude - ref=case		
chr27	19094866	A				#N/A	#N/A	exclude - ref=case		
chr27	19095700	T				0,555555556	0,333333333	exclude - variation in pools		
chr27	19095863	C				#N/A	#N/A	exclude - ref=case		
chr27	19095877	A				#N/A	#N/A	exclude - ref=case		
chr27	19095901	C	T	yes		#N/A	#N/A	interesting- not variable in pools and perfect pattern	Not conserved (no corresponding base in human, A in mouse/rat)	not conserved in human
chr27	19095978	A	G	yes	yes	#N/A	#N/A	in pools and perfect	human = A = controls	Interesting

chr27	19096053	T	A	yes		#N/A	#N/A	interesting- not variable in pools and perfect pattern	Human = - , rat = T	not conserved in human
chr27	19096199	T	G	yes	yes	0,181818182	0	Ref in non-atopic and	conservatin score	Interesting
chr27	19097058	T			yes but excl in QC	0	0	exclude - ref=case		
chr27	19097147	A			yes	0	1	exclude - ref=case		
chr27	19097445	A		yes		1	0,333333333	exclude - variation in pools		
chr27	19098565	A	G	yes		#N/A	#N/A	interesting- not variable in pools and perfect pattern	Human/mouse = G = cases...	Human/mouse equals GSD CAD cases
chr27	19099360	A	G	yes		0,6	0	interesting - match pools all the way?	Human/mouse/rat = G = cases...	Human/mouse/rat equals GSD CAD cases
chr27	19099698	C	T	yes		#N/A	#N/A	interesting- not variable in pools and perfect pattern	Human/mouse/rat = A (A most common, C in rabbit, pika, dog) cons=0.25	Interesting
chr27	19099734	G	A	yes	yes but excl in QC	0,125	0	interesting - match pools all the way?	Human/mouse/rat = -	No base in human/mouse/rat
chr27	19100895	C	T	yes		#N/A	#N/A	interesting- not variable in pools and perfect pattern	Human = C = controls, Mouse/rat = T=cases	Mouse/rat = GSD CAD cases
chr27	19101169	T		yes		#N/A	#N/A	interesting- not variable in pools and perfect pattern	Human/mouse/rat = - in a SINE	In SINE, no base in human/mouse/rat
chr27	19101299	A		yes		#N/A	#N/A	interesting- not variable in pools and perfect pattern	Human/mouse/rat = - in a SINE	In SINE, no base in human/mouse/rat
chr27	19101681	A		yes		#N/A	#N/A	interesting- not variable in pools and perfect pattern	Human/mouse = - in a SINE (rat=A)	In SINE, no base in human/mouse
chr27	19101711	C		yes		#N/A	#N/A	interesting- not variable in pools and perfect pattern	Human/mouse/rat = - in a SINE	In SINE, no base in human/mouse/rat
chr27	19101752	G		yes		0	1	exclude - variation in pools		
chr27	19102350	C		yes		0,428571429	0,333333333	exclude - variation in pools		
chr27	19102361	C	T	yes		#N/A	#N/A	interesting- not variable in pools and perfect pattern	Human/mouse/rat = T=cases	Human/mouse/rat = GSD CAD case
chr27	19102663	C	T	yes		#N/A	#N/A	interesting- not variable in pools and perfect pattern	Human = C = controls, Mouse/rat = T=cases	Human = C = controls but Mouse/rat = T=cases
chr27	19102681	T		yes	yes	0,666666667	0	exclude - common in other breeds...		

chr27	19102841	G		yes	yes	0,285714286	0	exclude - common in other breeds... and in LTR-repeat		
chr27	19103346	G	A	yes		#N/A	#N/A	interesting- not variable in pools and perfect pattern	Human/mouse/rat = A =cases and in an LTR-repeat	Human/mouse/rat = A =cases and in an LTR-repeat
chr27	19103635	C		yes		0,333333333	0,75	exclude - variation in pools		
chr27	19104423	C	T	yes		#N/A	#N/A	interesting- not variable in pools and perfect pattern	Human=C= controls, Mouse/rat=-, but T = dog, cat, ferret, panda, walrus, seal. Con=0.013	Case allele common in other species
chr27	19104717	G		yes		0,444444444	0,333333333	exclude - variation in pools		
chr27	19105411	A		yes		#N/A	#N/A	interesting- not variable in pools and perfect pattern	Human/mouse/rat = - in a SINE	In SINE, no base in human/mouse/rat
chr27	19105907	C		yes		#N/A	#N/A	interesting- not variable in pools and perfect pattern	Human/mouse/rat = - in a LINE	In LINE, no base in human/mouse/rat
chr27	19106099	T	C	yes		#N/A	#N/A	interesting- not variable in pools and perfect pattern	Human = G (not in dog), mouse/rat=-, In a LINE	In LINE, no base in mouse/rat (human = G, not in dog)
chr27	19107516	T		yes	yes	0,285714286	0	exclude - common in other breeds...		
chr27	19108039	A	G	yes		#N/A	#N/A	interesting- not variable in pools and perfect pattern	Human=-, Mouse/rat=A=controls	No base in human
chr27	19108790	T			yes	0,666666667	0	exclude - ref=case		
chr27	19109360	G	A	yes		#N/A	#N/A	interesting- not variable in pools and perfect pattern	Human=G=Controls, Mouse/rat=C, A = bat-species, cat and GSDcases	Case allele in bat species and cat
chr27	19109835	T	G	yes		#N/A	#N/A	interesting- not variable in pools and perfect pattern	Human/mouse=T=controls rat=-, but not possible to lift to human, T-repeat seq....	T repeat not mapped in human
chr27	19112076	C				0	0,083333333	exclude - ref=case & variation in pools		
chr27	19112099	C	G	yes		#N/A	#N/A	interesting- not variable in pools and perfect pattern	Human=G=cases, Mouse/rat=-	Human = GSD CAD case allele

chr27	19112169	A		yes	yes	#N/A	#N/A	interesting- top2 but not mapped in human - in repeat!	Human=-, in LTR-repeat	In LTR repeat, no base in human
chr27	19112352	C		yes		#N/A	#N/A	interesting- not variable in pools and perfect pattern	Human/mouse/rat=-, in a SINE	In SINE, no base in human/mouse/rat
chr27	19113531	A		yes	yes but excl in QC	#N/A	#N/A	interesting- not variable in pools and perfect pattern	Human/mouse/rat=-, in a LINE	In LINE, no base in human/mouse/rat
chr27	19114170	C		yes	yes	#N/A	#N/A	interesting- not variable in pools and perfect pattern	Human/dog/mouse/rat=C. Cons=1.1	Interesting
chr27	19114674	T		no		0	0	exclude - not perfect pattern...		
chr27	19115580	G		no		#N/A	#N/A	exclude - not perfect pattern...		
chr27	19116149	T		no		0	0	exclude - not perfect pattern...		
chr27	19117083	T		no		0,25	0,75	exclude - ref=case & variation in pools		
chr27	19117678	A		no		0,666666667	0	exclude - not perfect pattern...		
chr27	19118236	C	T	yes		#N/A	#N/A	interesting- not variable in pools and perfect pattern	Human =T = cases, Mouse/rat=-	Human = GSD CAD case
chr27	19118521	T	C	yes		#N/A	#N/A	interesting- not variable in pools and perfect pattern	Human=-, in a SINE	In SINE, no base in human
chr27	19119028	A	G	yes		0	0,333333333	exclude - rvariation in pools		
chr27	19119166	G	A	yes		#N/A	#N/A	interesting- not variable in pools and perfect pattern	Human=-, in a simple repeat	In simple repeat, no base in human
chr27	19119963	C	T	yes	yes	#N/A	#N/A	exclude - common in other breeds...		
chr27	19120627	A	G	yes		0	0	interesting - match pools all the way?	Human/Mouse/Rat= A=cases	Human/Mouse/Rat=A=cases
chr27	19121029	G	A	yes		0,769230769	0,363636364	exclude - variation in pools		
chr27	19121159	G	A	yes	yes but excl in QC	#N/A	#N/A	interesting- not variable in pools and perfect pattern	Human/Mouse/rat=G . A in baboon, cat, alpaca, camel... but TF-binding of JunD!! In 5'UTR	Human/Mouse/rat=G. A in baboon, cat, alpaca, camel... but TF-binding of JunD!! In 5'UTR
chr27	19121205	T		no	yes	#N/A	#N/A	exclude - common in other breeds...		

chr27	19121767	A		no		#N/A	#N/A	interesting- not variable in pools and perfect pattern		
chr27	19122037	T		no		0,6	0,6	exclude - not perfect pattern & variation in pools		
chr27	19122087	A		no		0	0,571428571	exclude - not perfect pattern & variation in pools		
chr27	19122222	T		no		1	0	exclude - not perfect pattern...		
chr27	19122404	G		no		0	0,4	exclude - not perfect pattern & variation in pools		
chr27	19122579	G		no		0,4	0,2	exclude - not perfect pattern & variation in pools		
chr27	19122692	C		no		0,333333333	0,5	exclude - not perfect pattern & variation in pools		
chr27	19122817	T	C	yes		0,333333333	0,25	exclude - variation in pools		
chr27	19123268	A	G	no		0,142857143	0,25	exclude - not perfect pattern & variation in pools		
chr27	19124099	A	T	yes		#N/A	#N/A	interesting- not variable in pools and perfect pattern	Human/mouse/rat=-	Human/mouse/rat=-
chr27	19124103	T	A	yes		#N/A	#N/A	interesting- not variable in pools and perfect pattern	Human/mouse/rat=-	Human/mouse/rat=-
chr27	19124996	A	T	no	yes	#N/A	#N/A	exclude - common in other breeds...		
chr27	19126533	C	T	yes	yes	0,571428571	0	interesting- top SNP but westiehap drops here...?!	T=human=case. T is common! In LTR-repeat!	T=human=case. T is common! In LTR-repeat!
chr27	19126638	T	A	yes	yes	0,5	0,4			
chr27	19127061	T	C	yes		0,363636364	0,090909091			
chr27	19127315	A	G	yes		#N/A	#N/A	interesting- not variable in pools and perfect pattern	Human/mouse/rat=-.	Human/mouse/rat=-.
chr27	19127748	A	G	yes	yes but excl in QC	#N/A	#N/A	interesting- not variable in pools and perfect pattern	Human = T, mouse/rat=-. T and C in most species. Only dog with A. Cons=-0.1	Human = T, mouse/rat=-. T and C in most species. Only dog with A. Cons=-0.1

chr27	19128370	A	G	yes		#N/A	#N/A	interesting- not variable in pools and perfect pattern	Human=G=cases, Mouse/rat=-	Human=G=cases, Mouse/rat=-
chr27	19128376	T	C	yes		#N/A	#N/A	interesting- not variable in pools and perfect pattern	Human/mouse/rat = - , in a LINE	Human/mouse/rat = - , in a LINE
chr27	19128442	G	A	yes		0,166666667	0,2			
chr27	19128578	A	T	yes	yes but excl in QC	0,3	0			
chr27	19130125	A	C	yes	yes	0,833333333	0,333333333			
chr27	19130510	A	T	no		#N/A	#N/A			
chr27	19130654	A	G	yes		0,444444444	0,181818182			
chr27	19131113	C	G	yes	yes	0,5	0			
chr27	19131298	A	G	yes		#N/A	#N/A	interesting- not variable in pools and perfect pattern	Human/mouse/rat = -	Human/mouse/rat = -
chr27	19131614	T	A	yes	yes	0	0			
chr27	19131849	T	A	yes		#N/A	#N/A	interesting- not variable in pools and perfect pattern	Human/mouse/rat = - , in a DNArepeat	Human/mouse/rat = -, in a DNArepeat
chr27	19131855	G	C	yes		#N/A	#N/A	interesting- not variable in pools and perfect pattern	Human/mouse/rat = - , in a DNArepeat	Human/mouse/rat = -, in a DNArepeat
chr27	19132336	G	A	yes		#N/A	#N/A	interesting- not variable in pools and perfect pattern	Human/mouse/rat = - , in a DNArepeat	Human/mouse/rat = -, in a DNArepeat
chr27	19132339	G	A	yes	yes	#N/A	#N/A	interesting- not variable in pools and perfect pattern	Human/mouse/rat = - , in a DNArepeat	Human/mouse/rat = -, in a DNArepeat
chr27	19133220	A	C	yes		#N/A	#N/A	interesting- not variable in pools and perfect pattern	Human/mouse/rat = - , in a DNArepeat	Human/mouse/rat = -, in a DNArepeat
chr27	19133235	A	G	yes		#N/A	#N/A	interesting- not variable in pools and perfect pattern	Human/mouse/rat = - , in a DNArepeat	Human/mouse/rat = -, in a DNArepeat
chr27	19135677	G	A	yes	yes	#N/A	#N/A	interesting- not variable in pools and perfect pattern	In a 1.9kb gap (300bp from one end) with SINEs and LINEs only present in dog. Risk allele - Not in other species. Confirmed in IGV	In a 1.9kb gap (300bp from one end) with SINEs and LINEs only present in dog. Risk allele - Not in other species. Confirmed in IGV
chr27	19135824	G	T	yes		#N/A	#N/A	interesting- not variable in pools and perfect pattern	Human/mouse/rat = - , in a LINE	Human/mouse/rat = - , in a LINE

chr27	19138377	C	T	yes		#N/A	#N/A	interesting- not variable in pools and perfect pattern	Human/mouse/rat = - , in a SINE	Human/mouse/rat = - , in a SINE
chr27	19138783	C	T	yes		0,666666667	0,1			
chr27	19139179	T	A	yes		#N/A	#N/A	interesting- not variable in pools and perfect pattern	Human/mouse/rat = - , in a SINE	Human/mouse/rat = - , in a SINE
chr27	19140837	T	G	no	yes	0,333333333	0		human = G = cases	human = G = cases
