# Supplementary material for: Multiple regulatory variants located in cell type-specific enhancers within the PKP2 locus form major risk and protective haplotypes for canine atopic dermatitis in German shepherd dogs
Source: BMC Genet. 2016 Jun 29;17:97. doi: 10.1186/s12863-016-0404-3 (PMC4928279; doi:10.1186/s12863-016-0404-3)
Supplement: Additional file 8: Table S8. — Summary of dogs genotyped for SNP 27:19,093,355. (PDF 39 kb) [file 12863_2016_404_MOESM8_ESM.pdf]

Table S8. Summary of dogs genotyped for SNP 27:19,093,355		
**Breed**	**No dogs in Taqman assay before QC**	**No dogs in finemapping (104SNPs) after QC**
American staffordshire terrier	1	
Beagle	15	
Bearded collie	16	
Bichon frisé	1	
Border collie	16	
Boxer	17	39
Bull terrier	8	19
Cairnterrier	12	
Chow-chow	2	
Dachshund	2	
Drever	1	
English springer spaniel	18	
Finnish lapphund	8	
Flatcoated retriever	17	
French bulldog	1	
German Shepherd dog	223	174
Giant schnauzer	16	10
Golden Retriever	326	25
Great dane	16	
Greyhound	1	
Hovawart	16	8
Irish setter	1	
Irish soft coated weaten terrier		2
Irish wolfhound	16	8
Jack russel		1
Japanese chin	1	
Labrador retriever	225	35
Leonberger	8	
Medium Poodle	1	
Miniature schnauzer	2	
Newfoundland	16	
Noca Scotia duck tolling retriev	24	
Norweigan elkhound		5
Polish lowland (pon)	8	
Poodle	16	
Poodle Toy	1	
Pug	1	
Puli	1	
Rottweiler	3	
Shar pei	16	
Shetland sheepdog	1	
Smalands hound		9
Standard poodle	2	
Swedish elkhound	16	
Wachtel		1
Welsh springer spaniel	17	
West Highland White Terrier	60	33
Whippet	6	
Mixed breed	5	1
