# Supplementary material for: Multiple regulatory variants located in cell type-specific enhancers within the PKP2 locus form major risk and protective haplotypes for canine atopic dermatitis in German shepherd dogs
Source: BMC Genet. 2016 Jun 29;17:97. doi: 10.1186/s12863-016-0404-3 (PMC4928279; doi:10.1186/s12863-016-0404-3)
Supplement: Additional file 10: Table S10. — Summary of additional breeds carrying the risk allele at SNP 27:19,093,355. (PDF 34 kb) [file 12863_2016_404_MOESM10_ESM.pdf]

Table S10. Summary of additional breeds carrying the risk allele at SNP 27:19,093,355

Breed	T/T	C/T	C/C	Total no of dogs	% of dogs carrying risk
Bearded Collie	0	5	11	16	31
Border Collie	1	4	11	16	31
English Springer Spaniel	3	3	10	16	38
Finnish lapphund	0	7	1	8	88
Giant Schnauzer	0	2	13	15	13
Nova Scotia duck tolling retreiver	6	9	7	22	68
Poodle	0	1	15	16	6
Welsh Springer Spaniel	1	9	6	16	63
Mongrel	0	1	4	5	20
