# Supplementary material for: Multiple regulatory variants located in cell type-specific enhancers within the PKP2 locus form major risk and protective haplotypes for canine atopic dermatitis in German shepherd dogs
Source: BMC Genet. 2016 Jun 29;17:97. doi: 10.1186/s12863-016-0404-3 (PMC4928279; doi:10.1186/s12863-016-0404-3)
Supplement: Additional file 11: Table S11. — Allele frequencies for all nine selected SNPs across all breeds. (PDF 50 kb) [file 12863_2016_404_MOESM11_ESM.pdf]

Table S11. Allele frequencies for all 9 selected SNPs across all breeds

ALL			CASES							CONTROLS							
Position	A1	A2	P.11	P.12	P.22	**non-carriers**	**carriers**	total	%dogs with risk	P.11	P.12	P.22	**non-carriers**	**carriers**	total	%dogs with risk	OR
18861228	C	A	81	79	24	**81**	**103**	184	56	92	65	25	92	90	182	49	1,3
19086778	C	T	108	56	18	**108**	**74**	182	41	131	44	4	131	48	179	27	1,9
19093355	C	T	122	45	11	**122**	**56**	178	31	156	19	5	156	24	180	13	3,0
19096199	T	G	122	51	12	**122**	**63**	185	34	154	22	5	154	27	181	15	2,9
19112169	A	G	123	52	12	**123**	**64**	187	34	155	22	5	155	27	182	15	3,0
19114170	C	G	119	52	12	**119**	**64**	183	35	152	22	5	152	27	179	15	3,0
19124996	T	A	48	66	73	**48**	**139**	187	74	83	54	45	83	99	182	54	2,4
19135677	G	A	132	45	10	**132**	**55**	187	29	163	15	5	163	20	183	11	3,4
19140837	G	T	87	54	**42**	**42**	**141**	183	77	60	43	76	76	103	179	58	2,5

BULLTERRIER			CASES							CONTROLS							
Position	A1	A2	P.11	P.12	P.22	**non-carriers**	**carriers**	total	%dogs with risk	P.11	P.12	P.22	**non-carriers**	**carriers**	total	%dogs with risk	OR
18861228	C	A	7	5	0	**7**	**5**	12	42	4	3	0	4	3	7	43	1,0
19086778	C	T	12	0	0	**12**	**0**	12	0	7	0	0	7	0	7	0	na
19093355	C	T	12	0	0	**12**	**0**	12	0	7	0	0	7	0	7	0	na
19096199	T	G	12	0	0	**12**	**0**	12	0	7	0	0	7	0	7	0	na
19112169	A	G	12	0	0	**12**	**0**	12	0	7	0	0	7	0	7	0	na
19114170	C	G	12	0	0	**12**	**0**	12	0	7	0	0	7	0	7	0	na
19124996	T	A	0	0	12	**0**	**12**	12	100	0	1	5	0	6	6	100	na
19135677	G	A	12	0	0	**12**	**0**	12	0	7	0	0	7	0	7	0	na
19140837	G	T	12	0	0	**0**	**12**	12	100	6	1	0	0	7	7	100	na

BOXER

Position	A1	A2	CASES P.11	P.12	P.22	non-carriers	carriers	total	%dogs with risk	CONTROLS P.11	P.12	P.22	non-carriers	carriers	total	%dogs with risk	OR
18861228	C	A	16	3	3	**16**	**6**	22	27	11	5	1	11	6	17	35	0,7
19086778	C	T	22	0	0	**22**	**0**	22	0	17	0	0	17	0	17	0	na
19093355	C	T	22	0	0	**22**	**0**	22	0	17	0	0	17	0	17	0	na
19096199	T	G	22	0	0	**22**	**0**	22	0	17	0	0	17	0	17	0	na
19112169	A	G	22	0	0	**22**	**0**	22	0	17	0	0	17	0	17	0	na
19114170	C	G	22	0	0	**22**	**0**	22	0	17	0	0	17	0	17	0	na
19124996	T	A	1	4	17	**1**	**21**	22	95	0	3	14	0	17	17	100	na
19135677	G	A	22	0	0	**22**	**0**	22	0	17	0	0	17	0	17	0	na
19140837	G	T	17	4	1	**1**	**21**	22	95	14	3	0	0	17	17	100	na

WHWT

Position	A1	A2	CASES P.11	P.12	P.22	non-carriers	carriers	total	%dogs with risk	CONTROLS P.11	P.12	P.22	non-carriers	carriers	total	%dogs with risk	OR
18861228	C	A	4	12	2	**4**	**14**	18	78	6	8	1	6	9	15	60	2,3
19086778	C	T	7	7	3	**7**	**10**	17	59	8	7	0	8	7	15	47	1,6
19093355	C	T	6	9	2	**6**	**11**	17	65	8	7	0	8	7	15	47	2,1
19096199	T	G	6	9	3	**6**	**12**	18	67	8	7	0	8	7	15	47	2,3
19112169	A	G	6	9	3	**6**	**12**	18	67	8	7	0	8	7	15	47	2,3
19114170	C	G	6	9	3	**6**	**12**	18	67	8	7	0	8	7	15	47	2,3
19124996	T	A	4	11	3	**4**	**14**	18	78	4	9	2	4	11	15	73	1,3
19135677	G	A	17	1	0	**17**	**1**	18	6	15	0	0	15	0	15	0	na
19140837	G	T	17	1	0	**0**	**18**	18	100	15	0	0	0	15	15	100	na

LABRADOR

Position	A1	A2	CASES							CONTROLS							
			P.11	P.12	P.22	**non-carriers**	**carriers**	total	%dogs with risk	P.11	P.12	P.22	**non-carriers**	**carriers**	total	%dogs with risk	OR
18861228	C	A	13	6	1	**13**	**7**	20	**35**	6	7	1	6	8	14	**57**	0,4
19086778	C	T	18	3	0	**18**	**3**	21	**14**	7	7	0	7	7	14	**50**	0,2
19093355	C	T	3	9	8	**3**	**17**	20	85	2	6	5	2	11	13	85	1,0
19096199	T	G	4	9	8	**4**	**17**	21	81	2	7	5	2	12	14	86	0,7
19112169	A	G	4	9	8	**4**	**17**	21	81	2	7	5	2	12	14	86	0,7
19114170	C	G	3	10	8	**3**	**18**	21	86	2	7	5	2	12	14	86	1,0
19124996	T	A	0	0	21	**0**	**21**	21	100	0	0	14	0	14	14	100	na
19135677	G	A	3	10	8	**3**	**18**	21	86	2	7	5	2	12	14	86	1,0
19140837	G	T	21	0	0	**0**	**21**	21	100	14	0	0	0	14	14	100	na

GOLDEN

Position	A1	A2	CASES							CONTROLS							
			P.11	P.12	P.22	**non-carriers**	**carriers**	total	%dogs with risk	P.11	P.12	P.22	**non-carriers**	**carriers**	total	%dogs with risk	OR
18861228	C	A	0	6	3	**0**	**9**	9	100	0	5	9	0	14	14	100	na
19086778	C	T	9	1	0	**9**	**1**	10	10	5	5	2	5	7	12	58	0,1
19093355	C	T	9	1	0	**9**	**1**	10	10	15	0	0	15	0	15	0	na
19096199	T	G	9	1	0	**9**	**1**	10	10	14	0	0	14	0	14	0	na
19112169	A	G	9	1	0	**9**	**1**	10	10	14	0	0	14	0	14	0	na
19114170	C	G	9	1	0	**9**	**1**	10	10	12	0	0	12	0	12	0	na
19124996	T	A	5	2	3	**5**	**5**	10	50	4	8	3	4	11	15	73	0,4
19135677	G	A	9	1	0	**9**	**1**	10	10	15	0	0	15	0	15	0	na
19140837	G	T	3	1	6	**6**	**4**	10	40	3	5	4	4	8	12	67	0,3

LRCADBREEDS

Position	A1	A2	P.11	P.12	P.22	non-carriers	carriers	total	%dogs with risk
18861228	C	A	41	23	15	**41**	**38**	79	48
19086778	C	T	75	3	1	**75**	**4**	79	5
19093355	C	T	79	0	0	**79**	**0**	79	0
19096199	T	G	79	0	0	**79**	**0**	79	0
19112169	A	G	79	0	0	**79**	**0**	79	0
19114170	C	G	79	0	0	**79**	**0**	79	0
19124996	T	A	22	20	37	**22**	**57**	79	72
19135677	G	A	79	0	0	**79**	**0**	79	0
19140837	G	T	40	18	21	**21**	**58**	79	73

GSD

			CASES							CONTROLS							
Position	A1	A2	P.11	P.12	P.22	non-carriers	carriers	total	%dogs with risk	P.11	P.12	P.22	non-carriers	carriers	total	%dogs with risk	OR
18861228	C	A	34	43	13	**34**	**56**	90	62	57	24	2	57	26	83	31	3,6
19086778	C	T	30	43	14	**30**	**57**	**87**	66	56	24	2	56	26	82	32	4,1
19093355	C	T	57	26	1	**57**	**27**	84	32	75	6	0	75	6	81	7	5,9
19096199	T	G	56	32	1	**56**	**33**	89	37	74	8	0	74	8	82	10	5,5
19112169	A	G	57	33	1	**57**	**34**	91	37	75	8	0	75	8	83	10	5,6
19114170	C	G	54	32	1	**54**	**33**	87	38	74	8	0	74	8	82	10	5,7
19124996	T	A	33	43	15	**33**	**58**	91	64	57	24	2	57	26	83	31	3,9
19135677	G	A	56	33	2	**56**	**35**	91	38	75	8	0	75	8	83	10	5,9
19140837	G	T	14	43	30	**30**	**57**	87	66	2	24	56	56	26	82	32	4,1
