# Supplementary material for: Multiple regulatory variants located in cell type-specific enhancers within the PKP2 locus form major risk and protective haplotypes for canine atopic dermatitis in German shepherd dogs
Source: BMC Genet. 2016 Jun 29;17:97. doi: 10.1186/s12863-016-0404-3 (PMC4928279; doi:10.1186/s12863-016-0404-3)
Supplement: Additional file 12: Table S12. — Phase results of nine SNPs across all breeds. (PDF 32 kb) [file 12863_2016_404_MOESM12_ESM.pdf]

Table S12.Phase results of nine SNPs across all breeds.

HAPLOTYPE	VARIANTS	NUMBER OF ALLELES IN ALL BREEDS	NUMBER OF ALLELES IN GSD ONLY
1	CCCTACAGG	129	0
2	CCCTACAAG	1	0
3	CCCTACAAT	1	1
4	CCCTACTGG	22	0
**5**	**CCCTACTGT**	**297**	**244**
6	CCCTACTAT	1	1
7	CCCTAGAGG	1	0
8	CCTGGGAAG	33	0
9	CTCTACAGG	4	2
10	CTCTACTGT	1	0
11	CTTGGGAGG	1	0
12	CTTGGGAAG	6	2
13	ACCTACAGG	58	0
14	ACCTACAGT	4	0
15	ACCTACTGG	6	0
16	ACCTACTGT	40	2
**17**	**ATCTACAGG**	**66**	**35**
18	ATCTACTGT	1	0
19	ATTGGGAGG	6	0
**20**	**ATTGGGAAG**	**48**	**41**
21	ATTGGGTGG	14	0
