# Supplementary material for: Multiple regulatory variants located in cell type-specific enhancers within the PKP2 locus form major risk and protective haplotypes for canine atopic dermatitis in German shepherd dogs
Source: BMC Genet. 2016 Jun 29;17:97. doi: 10.1186/s12863-016-0404-3 (PMC4928279; doi:10.1186/s12863-016-0404-3)
Supplement: Additional file 13: Table S13. — DNA fragments and primers used for cloning with the luciferase reporter vector pGL4.26. (PDF 27 kb) [file 12863_2016_404_MOESM13_ESM.pdf]

Table S13. DNA fragments and primers used for cloning with the luciferase reporter vector pGL4.26.

SNP ID	forward primer	reverse primer	amplicon length	cloning sites	sequence
**27:18861228**	AGACTGATGGAAGATGGCTTGCT	TAGCCAAGCTTCAGCCTATCAGGGTATTGTTCTG	318 bp	EcoRV+HindIII	AGACTGATGGAAGATGGCTTGCTTACCCAAGCTACTCTGGGTCTTCCTCTCTTAACCACAACCTCTGCAACACCTGTAGT/CGCCCCTTGTCTGTGTAGCTATTCAAGCAAGGTATCAGCCTGCTTAGCAGATCAAGCAAGTAATATCCATACTGAAGTCCTATGCTGCTAATGTATTGACTTTCTCACTCCACACTACTCAC**[A/C]**AAGCAGACTAGACAACAAAAGGTAAGTCACCTCTACACCTGGTATGT/CGCCTGAAGGACAACAACAATTTTAATTCCTGCATCAAGCAATCCTCAGAACAATACCCTGATAGGCTG
**27:19086778**	TACAATGAAATATGTTAGAGCAGTG	TAGCCAAGCTTGATGTAATACGGTGAGGCAATG	517 bp	EcoRV+HindIII	TACAATGAAATATGTTAGAGCAGTGGCCCCTGAAGCATTAACATCTGCATTACCTAGAAGTTGTTAGGATCCTCAGGTCCTACCTCAGACCT/GGAATCAGAAACTCTCAGGGTGGGGCTGAGCAATCTAGTTTTAACAAGCCCTCCAGGTGATTCTGATGCAGCTGAATTGTGAGAGGCCTGATGTCAGAGGTACAAGTGAAGTGCTGCAGGAATGCAGAACAAAGAACCATTCATGCC**[C/T]**GCTGAGTGGGATCAGAAAGACACTACAGACTAGGTGCCAAATGAACTAGGCCTTGGTGGATTATTTCAATAGTCAGAAATTCTCTAAGGACTAAGGACTAACTCCTTGAACAGCAGAAGAGGTGCCTAAATGGTCTCTTTGAATCCATCTTCTGTACTGTTCACTAGAACTGCGTTTTCATTGTGCTTTTGATCTAATAACACATTTTGATCTATTTAAAAGTATTTTGCATCTCAAAATTTTCTCTGTAGCTCCCATTGCCTCACCGTATTACATCA
**27:19093355**	GTAAATTTAAAAGATGTAAATGGTAT	TAGCCAAGCTTAGTCCTTTTCATGTATCCTTAATA	479 bp	EcoRV+HindIII	GTAAATTTAAAAGATGTAAATGGTATTTAAATAGATATATAAAATCTTGGTTTTAAAAAGTGATTGGTTTAATAAGCTAAATCTCAGGGGTTT(+T)AAATGATCAGTTTGAAATCTCTCAATAGTAATTAAGTTAGTAAAACAAATAAATCAAACATCAAACCACACAAATATGGCTATGCTTA**[C/T]**CTGGTCACCCCTCTCTGATATCCCTGAACCCAGTATTCTTAAGTGACTAGGTTGCATTAGAATTGTCCAATTCTACAACCATTGCAAAGGTCTCAGTTCTTCAGACTAAATTTTCTGCTGTCCTATTCGTGCCTTTAGCAGATAGGCCCAGATTAAGAGTTGTACTTCTCAATGATTATAATGACAGACCTCTTTGCCAGGGAAGCTCTTTATTCTTATCAATCAACTCA/GTAAATACGGACTACCTGCTTTGGTCTCAGCCCTGGGATAATTATTAAGGATACATGAAAAGGACTA
**27:19096199**	TAGACAGGTACCATTTCCCCAAGCTAGTTGTCCGTA	GATAAGAAGAACTGCAATGTAGTGT	324 bp	KpnI+EcoRV	ATTTCCCCAAGCTAGTTGTCCGTACACCACACACCAAGCTTTGGACATTCTGTGTTGTAACAAACCTTCTTTCAGTGCTGGCAAACTAGCTATAACAGCATATCTGCA[**G/T**]GTAGCCACTCACAGATAACTTTTTATGGAAGGGCCAATAAAAGCCCTATTAGAAAGAGGTAATTATGATTTTAAACAAGAGTCACAGGTCAGGATTCCAAGTGAGATTTTATCATGCCATAATTTAAAGAATATTGTGACAATTACAGATAGCAGTATTTGTAAAGTGCCTACTATCACATCCATCACACACACTACATTGCAGTTCTTCTTATC
**27:19112169**	ATTTCCCTTGTTGTAACATGTGTG	TAGCCAAGCTTGAGGGCCACTAAAATGACAAACTG	211 bp	EcoRV+HindIII	ATTTCCCTTGTTGTAACATGTGTGTAGCATGCATGTGACATTGTGTAAC/GATATATAACATGTGTTTTGCCAC/GTAGCCTGTAAAAAGATAAAGTGGAAAGAAACAAAGAAAGGAAAGAAGAAAGAGAAGGAAGGGAGAAAGG[**G/A**]AGAGAGGGAAAGCGAGAGGAAAATTTCTAT(+T)CCAATGTCCATATTCCAGTTTGTCATTTTAGTGGCCCTC
**27:19114170**	GTAGTCCCATGCTGTCATAAGATG	TAGCCAAGCTTGTGAGGATTTCATTCCAAGTCAG	266 bp	EcoRV+HindIII	GTAGTCCCATGCTGTCATAAGATGATCCACTAATAAAGCTCCTCTCATTTTAAGGACAGTATTGAGAAGAGGCTTCCG[**C/G**]AAGATAGATGCAATTTTTAAATTCCAAATTGGGTTTATATATAATTTGAGTGAAAGGGCCCATCTTGATTCAGAGTATTAATAGC/ACAGGCATAAATGAAAATGAGTTTGAAATACATCAATCGTGTTTCTCCTGAGAGACTTCTAGACTAAAAGTTTCCATGTGCTGACTTGGAATGAAATCCTCAC
**27:19135677**	GCACCCCTGTACTAAGAATATTTTGTAG	TAGCCAAGCTTGAAAAGAGTAGTATTGAAAGGGTTTG	164 bp	EcoRV+HindIII	GCACCCCTGTACTAAGAATATTTTGTAGGAAAATATGACAA/GTTGGTTATCTCAGTGTCAACATAAGCCAACATATCCCCAAAATAC[**G/A**]TGGATTATGTTAAGTTTATGCCATTTTTGAGCATGGCCAAACCCTTTCAATACTACTCTTTTC
**27:19140837**	CCCTGTTCAAGATGAGAAAGCTGA	TAGCCAAGCTTAGCTAAGGCCATTGCACGCTGA	406 bp	EcoRV+HindIII	CCCTGTTCAAGATGAGAAAGCTGAAACAATATGGCAAAGGAACTTGCTCAATGTGACAGCCAGAAAGAATCAAACTGTGATTCAAGACTATATTTGTCTTACTCCAAAGCCTGTATTTTTACTGCACTCTGTGGCTCCCCTGACTCAGAGAGACTATAATCAGGACCCTCAGAAGGCTAGTGAAGCTGCTAGACCAGGACAAAG**[T/G]**CTGC/TGGGGGTTAGCATGCTCCAACTCTCAGAGAGAGGAAGCACATGGTTATCATGACTTGCAACAGCAAAGCAGATATTAAGATGCAACTCCTTGCCAGAGGCTCTCTGCAAAATTCAAACTGTGGGTGAGCAGATCAAAGTGAAAAAGGCCCTAGGACTAAGATCATGGATAAGAGGTCAGCGTGCAATGGCCTTAGCT
