# Supplementary material for: Multiple regulatory variants located in cell type-specific enhancers within the PKP2 locus form major risk and protective haplotypes for canine atopic dermatitis in German shepherd dogs
Source: BMC Genet. 2016 Jun 29;17:97. doi: 10.1186/s12863-016-0404-3 (PMC4928279; doi:10.1186/s12863-016-0404-3)
Supplement: Additional file 16: Table S16. — Additional SNPs in LD with the two top GWAS SNPs in GSDs selected for genotyping in the 122 SNP set. (PDF 38 kb) [file 12863_2016_404_MOESM16_ESM.pdf]

Table S16. Additional SNPs in LD with the two top GWAS SNPs in GSDs selected for genotyping in the 122 SNP set

chromosome	position	dog ref	alt allele	comments	ref	alt allele	T1	T2	T6	T7	T8	7=perfect pattern
chr27	18457482	T	C	around second little peak, cons region d-h but not base	T	C	T	T	C	C	C	7
chr27	18459109	T	C	around second little peak, cons and match GSD (not pools…)	T	C	T	T	C	Y	Y	7
chr27	18459330	C	T	around second little peak, cons and match GSD (not pools…)	C	T	C	C	T	Y	Y	7
chr27	18486358	G	A	around second little peak, cons and match GSD (not pools…)	G	A	G	G	A	R	R	7
chr27	18486849	A	G	around second little peak, fairly cons and match gsd	A	G	A	A	G	G	G	7
chr27	18490669	A	G	around second little peak, fairly cons and match gsd	A	G	A	A	G	G	G	7
chr27	18507811	C	T	around second little peak, fairly cons and match gsd	C	T	C	C	T	Y	Y	7
chr27	18540760	C	T	around second little peak, fairly cons and match gsd	C	T	C	C	T	Y	Y	7
chr27	19143309	G	A	Around peak at 19.143-19.146Mb	G	A	G	G	A	A	A	7
chr27	19143986	G	A	Around peak at 19.143-19.146Mb	G	A	G	G	A	R	R	7
chr27	19145949	A	G	Around peak at 19.143-19.146Mb	A	G	A	A	G	R	R	7
chr27	19146102	C	T	Around peak at 19.143-19.146Mb	C	T	C	C	T	Y	Y	7
chr27	19146391	T	C	Around peak at 19.143-19.146Mb	T	C	T	T	C	Y	Y	7
chr27	19146541	A	G	Around peak at 19.143-19.146Mb	A	G	A	A	G	R	R	7
chr27	19170561	C	T	extra SNPs outside the PKP2 region	C	T	T	T	C	Y	Y	7
chr27	19177149	C	T	extra SNPs outside the PKP2 region	C	T	T	T	C	Y	Y	7
chr27	19182732	G	A	extra SNPs outside the PKP2 region	G	A	A	A	G	R	R	7
chr27	19197711	G	T	extra SNPs outside the PKP2 region	G	T	T	T	G	K	K	7
chr27	19298550	T	C	extra SNPs outside the PKP2 region	T	C	T	T	C	Y	Y	7
chr27	19299519	A	G	extra SNPs outside the PKP2 region	A	G	A	A	G	R	R	7
